# Remarkable confusion in some Western Palearctic *Clepsis* leads to a revised taxonomic concept (Lepidoptera, Tortricidae)

**DOI:** 10.3897/zookeys.885.38655

**Published:** 2019-11-04

**Authors:** Boyan Zlatkov, Peter Huemer

**Affiliations:** 1 Institute of Biodiversity and Ecosystem Research, Bulgarian Academy of Sciences, 1 Tsar Osvoboditel Blvd., 1000 Sofia, Bulgaria Institute of Biodiversity and Ecosystem Research, Bulgarian Academy of Sciences Sofia Bulgaria; 2 Tiroler Landesmuseen Betriebsges.m.b.H., Naturwissenschaftliche Sammlungen, Krajnc-Str. 1, A-6060 Hall, Austria Tiroler Landesmuseen Betriebsges.m.b.H. Hall Austria

**Keywords:** DNA barcoding, *Clepsis consimilana*, *C. neglectana*, genitalia, vesica

## Abstract

The taxonomy of some Palearctic species of the genus *Clepsis* Guenée, 1845 (Lepidoptera, Tortricidae), in particular *C. neglectana* sensu auctt. and *C. consimilana* sensu auctt., is revised based on combined characters of external and internal adult morphology, including everted vesicae in male genitalia, and DNA barcodes. *Clepsis striolana* (Ragonot, 1879), **stat. rev.**, *C. acclivana* (Zerny, 1933), *C. trivia* (Meyrick, 1913), **stat rev.**, and *C. xylotoma* (Meyrick, 1891), **stat. rev.** are resurrected from synonymy with *C. neglectana* (Herrich-Schäffer, 1851), *C. semiana* (Chrétien, 1915), **stat. nov.** is considered as a valid species; *C. eatoniana* (Ragonot, 1881), **stat. rev.**, is resurrected from synonymy with *C. consimilana* (Hübner, 1817), and *C. razowskii* Gastón, Vives & Revilla, 2017, **syn. nov.** is synonymised with *C. eatoniana*.

## Introduction

*Clepsis* Guenée, 1845 is a large genus in the tribe Archipini with 163 valid species described worldwide (Gilligan et al. 2014) and 32 species present in Europe (Aarvik 2013). It is likely that these numbers will change in the future because a recent phylogenetic study revealed that the genus is paraphyletic and its position within the tribe is not completely resolved (Dombroskie and Sperling 2013). As such, the genus is in need of revision and the status of some taxa assigned to the genus should be evaluated. As with many groups of Lepidoptera, characters of the male genitalia have been used almost exclusively to define the present-day species boundaries of the taxa comprising *Clepsis*. Many taxa were synonymised in the past decades because of their similarity in male genitalia, resulting in long synonymic lists for some species. Two of the most problematic species are *C. consimilana* (Hübner, 1817) and *C. neglectana* (Herrich-Schäffer, 1851), each with many synonymic taxa: 13 for *C. consimilana* and eight for *C. neglectana* (Gilligan et al. 2014). These species are widely distributed in the Western Palaearctic and parts of Asia (Razowski 2002), and appeared to be variable in wing pattern, size, and even genital morphology. DNA barcode sequences of these species from different parts of Europe revealed that there is a considerable genetic variability within each of the species, and the DNA sequences cluster into compact groups. These groups appear to correspond well with certain wing patterns, secondary sex characters (costal fold in males), and genital morphology. It appears that some previously described taxa in this group were erroneously synonymised because of an incorrect assessment of variability in the genitalia, and that these names should be reinstated. A few taxa (also previously synonymised) were described from a single male specimen and no further material has been collected. Their present status is unknown and these taxa are in need of thorough revision, which is currently not feasible because of difficulties in obtaining fresh material from their type localities. The purpose of the present paper is to revise some of the taxa previously in synonymy with *C. neglectana* and *C. consimilana*, and to discuss some questionable taxa.

## Materials and methods

We examined ca. 150 specimens from various European collections. Data for the specimens and their repository are given in the results for each taxon treated. The genitalia were dissected and mounted following the methods of Robinson (1976) and Zlatkov (2011). The everted vesicae were drawn before mounting on a slide as described by Zlatkov and Huemer (2017). The female genitalia were submerged in euparal essence without deformation and drawn in a manner similar to the male genitalia. A three-dimensional perception of the studied structures was achieved by modifying a compound microscope Amplival (Carl Zeiss Jena) after Hammond (1996). Certain structures whose shape and size are not affected by preparation were measured. Angles were measured using a photograph of a protractor aligned digitally to photos, and the linear measurements were performed using ocular scales. Forewing length was preferred rather than wingspan, as the latter depends too much on the condition of the specimen. The terminology of the wing pattern and genitalia follows Kuznetzov (1978) and Razowski (2002). The classification of cornuti and microstructures of the signa are after Anzaldo et al. (2014) and Lincango et al. (2013), respectively.

DNA barcode sequences of the mitochondrial COI gene, a 658 base-pair long segment of the 5’ terminus of the mitochondrial COI gene (*cytochrome c oxidase 1*), were obtained from 15 specimens, supplemented by 23 publicly available sequences in BOLD (Table 1). DNA samples from dried legs were prepared according to prescribed standards using the high-throughput protocol of deWaard et al. (2008). Samples were processed at the Canadian Centre for DNA Barcoding (CCDB, Biodiversity Institute of Ontario, University of Guelph). DNA sequencing resulted in a full barcode for 36 specimens supplemented by nine sequences longer than 550 bp. Thirty-one of the sequences belong to individuals in the *Clepsis consimilana* species group and 14 of the sequences represent individuals in the *Clepsis neglectana* species group. Details of successfully sequenced voucher specimens (Table 1), including complete geographic data and images, can be accessed in the Barcode of Life Data Systems (BOLD; Ratnasingham and Hebert 2007) in the public dataset “*Clepsis consimilana* – *Clepsis neglectana* species groups” https://doi.org/10.5883/DS-Clepsis. All newly generated sequences were submitted to GenBank.

**Table 1.** List of successfully sequenced specimens of the *C. consimilana* and *C. neglectana* species-groups.

Species	Country/Ocean	Sample ID	Process ID	BIN
*C. consimilana*	Austria	TLMF Lep 22912	LEAST314-17	BOLD:AAC4212
	Austria	TLMF Lep 18827	LEATJ967-15	BOLD:AAC4212
	Austria	TLMF Lep 17661	LEATI276-15	BOLD:AAC4212
	Austria	TLMF Lep 09912	PHLAW115-13	BOLD:AAC4212
	Bulgaria	CCDB-11133-B10	BTLBP212-11	BOLD:AAC4212
	Bulgaria	CCDB-11133-B08	BTLBP210-11	BOLD:AAC4212
	Canada	PFC-2006-1202	LPVIA896-08	BOLD:AAC4212
	Canada	PFC-2006-1201	LPVIA895-08	BOLD:AAC4212
	Canada	PFC-2006-0914	LPVIA672-08	BOLD:AAC4212
	Canada	PFC-2006-0917	LPVIA675-08	BOLD:AAC4212
	Canada	PFC-2006-2539	LPVIB987-08	BOLD:AAC4212
	Canada	PFC-2006-1560	LPVIB188-08	BOLD:AAC4212
	Canada	PFC-2006-1559	LPVIB187-08	BOLD:AAC4212
	Canada	PFC-2006-1203	LPVIA897-08	BOLD:AAC4212
	Croatia	TLMF Lep 23752	LEAST1249-17	BOLD:AAC4212
	Denmark	MM19630	LEEUA222-11	BOLD:AAC4212
	Germany	BC ZSM Lep 27847	FBLMU817-09	BOLD:AAC4212
	Germany	BC ZSM Lep 25760	FBLMU250-09	BOLD:AAC4212
	Germany	BC ZSM Lep 25551	FBLMU041-09	BOLD:AAC4212
	Germany	BC ZSM Lep 23362	FBLMT132-09	BOLD:AAC4212
	Italy	TLMF Lep 15577	LEATH365-14	BOLD:AAC4212
	Italy	TLMF Lep 10273	LEATB096-13	BOLD:AAC4212
	Italy	TLMF Lep 02195	PHLAC160-10	BOLD:AAC4212
	United Kingdom	UKLB40C05	CGUKD697-09	BOLD:AAC4212
	United Kingdom	UKLB34D07	CGUKD152-09	BOLD:AAC4212
	United Kingdom	UKLB30C02	CGUKC754-09	BOLD:AAC4212
	United Kingdom	UKLB22A10	CGUKB985-09	BOLD:AAC4212
	United Kingdom	UKLB20D10	CGUKB831-09	BOLD:AAC4212
	United States	RWWA-3675	RWWC1018-12	BOLD:AAC4212
*C. eatoniana*	Spain	TLMF Lep 03187	PHLSA012-11	BOLD:AAJ1025
	Spain	TLMF Lep 03186	PHLSA011-11	BOLD:AAJ1025
*C. neglectana*	Finland	MM18257	LEFIK682-10	BOLD:AAM0282
	Finland	MM15659	LEFIG795-10	BOLD:AAM0282
*C. striolana*	Italy	TLMF Lep 18324	LEATJ749-15	BOLD:AAM0282
*C. trivia*	Greece	TLMF Lep 16958	LECRT048-15	BOLD:ACT3810
	Greece	TLMF Lep 16957	LECRT047-15	BOLD:ACT3810
	Greece	TLMF Lep 16956	LECRT046-15	BOLD:ACT3810
	Greece	TLMF Lep 16953	LECRT043-15	BOLD:ACT3810

Degrees of intra- and interspecific variation in the DNA barcode fragments were calculated under the Kimura 2 parameter (K2P) model of nucleotide substitution using analytical tools in BOLD Systems v. 4.0 (http://www.boldsystems.org). A neighbour-joining tree of DNA barcode data of selected taxa was constructed using Mega 6 (Tamura et al. 2013) (Fig. 16).

Institutional acronyms:

**BMNH** Natural History Museum, London, United Kingdom

**CJJ** Private collection of Jari Junnilainen, Vantaa, Finland

**CWK** Private collection of Wojciech Kubasik, Poznań, Poland

**MFN** Museum für Naturkunde, Berlin, Germany

**MNHN** Muséum National d’Histoire Naturelle, Paris, France

**MTD** Senckenberg Museum für Tierkunde, Dresden, Germany

**NHMW** Naturhistorisches Museum Wien, Austria

**NMNHS** National Museum of Natural History, Sofia, Bulgaria

**SDEI** Senckenberg Deutsche Entomologisches Institut, Müncheberg, Germany

**TLMF** Tiroler Landesmuseum Ferdinandeum, Innsbruck, Austria

**ZMUO** Zoological Museum, University of Oulu, Finland

**ZSM** Zoologische Staatsammlung München, Germany

## Results

### Morphology of genitalia

The male genitalia of the studied species, especially the valva, are relatively complex. The female genitalia are simple, but there is some discrepancy in the terms used in literature. For the purpose of unification, we provide general schemes of male and female genitalia of the studied taxa.

**Figure 1.**
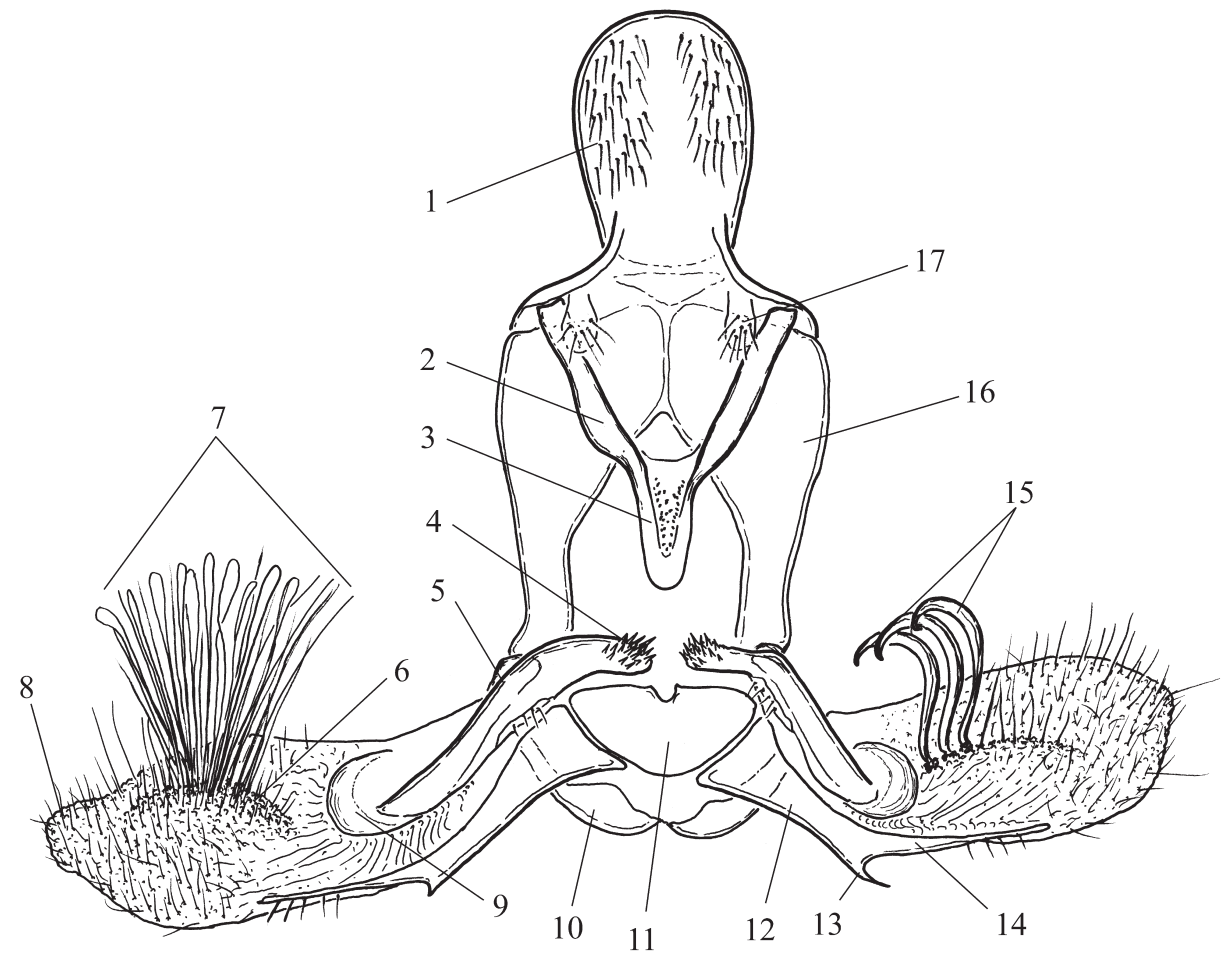
Scheme of male genitalia of *Clepsis neglectana* and *C. consimilana* species groups **1** uncus **2** arm of gnathos **3** medial part of gnathos **4** labis **5** costal sclerite **6** membranous medial protuberance **7** tuft of scales and setae **8** brachiola **9** hemispherical protrusion **10** vinculum **11** juxta **12** basal part of sacculus **13** saccular process **14** distal part of sacculus **15** modified setae **16** tegumen **17** socius.

**Male** (Fig. 1). The uncus is a large wide plate, convex dorsally, densely setose on both dorsal and ventral surfaces. In some species its shape can vary depending on the amount of pressure applied during preparation of the slide, and thus should be interpreted with caution. The gnathos is large, with a relatively large medial part, plough-shaped in all taxa. Because it projects posteriorly, it is often bent to the left or right in microscope slides. The socius is small, membranous, setose, and teardrop-shaped. The valva is complex, with largely sclerotised basal half and membranous distal half. The costa is enlarged, swollen, with a large more or less z-shaped, tubular costal sclerite. The sclerite protrudes medially into a subspherical or elongated spinulate structure called labis (plural labides) (Razowski 1979) or processus basalis (Horak 1984). The labides are probably derivatives of the transtilla but since they are not connected with a median sclerite, the term transtilla is less appropriate in this case. They are covered with large sclerotised acanthae. The middle part of the costal sclerite medially transforms into a setose membrane. The ventral part of the costal sclerite ends into a hemispherical transparent protrusion on the medial surface of the valva surrounded by a wrinkled furrow. The sacculus is large, medially concave, sclerotised, with a large triangular basal half, an elongated apical half and a ventrally pointed process located at the transition between the two halves. The distal part of the valva is membranous and terminates into a rounded, more or less extended plate referred to as a brachiola. The medial surface of the membranous part of the valva is densely covered by setae and scales forming a tuft on a small protuberance in some species. Most of the scales can be easily removed during cleaning of the macerated genitalia, but they should not be confused with a tuft of firmly attached scales and setae in some species which is well preserved after such manipulations. There can be a row of several very large modified setae on the median surface of valvae. The setae are elliptical in cross-section, falcate, with curved tips and can be broken during preparation of genitalia, and perhaps during copulation (males with setae broken off were found), but their sockets still remain observable (Fig. 7F). Apart from the medial scales and setae, there is a large, membranous pad on the lateral (“external”) surface of the valva bearing a tuft of long deciduous scales (possibly androconia), which are usually lost during dissection. The juxta is a wide nearly semi-circular plate with a median incision for articulation with the caulis. The phallus is slightly sinuate, pointed, predominantly membranous dorsally, with a long stout lateral process on the left side. The anterior portion of the phallus is bent at a large ventral angle. The vesica is comparatively simple, tubular, with a small basal widening and a thumb-like diverticulum apically. A few long aciculate deciduous cornuti are attached near the base of the diverticulum, adjacent to the gonopore.

**Female** (Fig. 8). The papillae anales and the apophyses are not specialised and they are similar in all the taxa studied. The sterigma is more-or-less broadly funnel-shaped, dorso-ventrally flattened, sclerotised, with a large excavation on the dorsal wall. It ends into a narrow, funnel-shaped colliculum comprising areas of sclerotised tanned plicate and thickened transparent cuticle. This part is referred to as antrum by some authors (Kuznetzov 1978), but the latter term is used mainly for the sclerotised ring or collar in Cochylini (Razowski 1970). Both structures are localised in the same region and are probably homologous (Horak 1984). The ductus seminalis is inserted at the transition between the colliculum and the ductus bursae. The ductus bursae is relatively long, with a smooth elongate sclerite referred to as cestum extending on most of its length and sometimes expanding onto the corpus bursae as well. The corpus bursae is ovoid with two types of signa: one plate-like consisting of sclerotised papillae, and a hook-shaped one that is more typical of most Archipini with longitudinal rows of teeth and a large capitulum.

### Taxonomy

#### The *Clepsis neglectana* species group

The species in this group externally are similar to taxa in the *C. consimilana* group but are distinguished by the absence of large modified setae on the median surface of the valvae (Fig. 1).

##### Clepsis neglectana

Taxon classificationAnimaliaLepidopteraTortricidae

(Herrich-Schäffer, 1851)

4C5527E9-71DC-5CF3-B585-B37C9E7A9A9A

neglectana Herrich-Schäffer, 1847 (uninominal): pl. 9, fig. 59
Tortrix (Lozotaenia) neglectana Herrich-Schäffer, 1851: 167 (Germany)
Tortrix (Heterognomon) betulifoliana Lederer, 1859: 248 (Poland)
Cacoecia delibatana Rotschild, 1912: 27 (Hungary)
Tortrix dorana Kennel, 1919: 60 (Kazakhstan: Ili)
Tortrix flavana Duponchel, 1834: 87, pl. 239, fig. 6 (France)
non Tortrix striolana Ragonot, 1879
non Tortrix xylotoma Meyrick, 1891
non Tortrix trivia Meyrick, 1913
non Cacoecia acclivana Zerny, 1933

###### Material examined.

Lectotype ♂ (here designated): pinned, genitalia on a slide, with 7 labels: “Typus!” [handwritten] “Origin.” [red printed] “ex coll. 1/1 / Staudinger” [printed] “Dresden / n. sp.” [handwritten] “Genital-Unters. / Nr. 0024” “H.-Sch.” [both printed] “Lectotype / *Tortrix neglectana* / Herrich-Schäffer, 1851 / des. Zlatkov & Huemer 2019” [red printed].

GERMANY • 1 ♂; Dresden; Staudinger leg.; GS (genitalia slide) 0024; MFN.

Other material: GERMANY •1 ♂; Disque leg.; GS M.044; ZSM • 1 ♂; Southern Germany; Disque leg.; GS 1/2.11.2018; ZSM • 1 ♂; Dresden; Schmidt leg.; GS 1/5.12.2018; MTD • 1 ♀; Dresden, Loschwitz; GS 3/4.12.2018; MTD • 1 ♀; Dresden [?];6 Jul. 1897; GS 4/4.12.2018; MTD • POLAND • 1 ♂; Szczecin, Dabie; 30 Jul.; unknown leg.; GS 1/5.11.2018; ZSM • FINLAND • 2 ♂♂; Valkeala; 28 Jul.–4 Aug. 1999; T. Mutanen leg.; GS 1/2.2.2018, 2/9.2.2018; ZMUO • 1 ♂; Valkeala; 22–27 Jul. 1999; GS 1/7.11.2018; ZMUO • 2 ♂♂; Valkeala; 20–24 Jul. 1998; P. Sundell & T. Mutanen leg.; GS 1/5.2.2018, 1/9.2.2018; ZMUO • 1 ♂; Haapasaari; 15 Jul. 1973; J. Jalava leg.; GS 1/3.2.2018; ZMUO • 2 ♀♀; Haapasaari; 6–7 Aug. 2004; J. Junnilainen leg.; GS 1/14.12.2018, 2/14.12.2018; CJJ.

###### Diagnosis.

Externally, *C. neglectana* is similar to the other species in the *C. neglectana* species group (apart from *C. striolana*) and *C. consimilana*, but the markings are darker and the costal fold is rudimentary. The male genitalia are very close to *C. striolana*, with the most obvious difference in the shape of labis; additionally, the setal tuft of the valva is less compact and smaller, the sacculus is straighter, and the vesica most often bears a single cornutus. *Clepsis neglectana* differs from *C. acclivana* and *C. trivia* by the shape of the uncus and numerous characters on the valvae and phallus. Apart from the forewing pattern, the female differs from *C. striolana* by the presence of a transparent protrusion of the colliculum on the right side, and from *C. trivia* by the length and shape of the colliculum. In contrast to *C. trivia*, both *C. neglectana* and *C. striolana* lack a plate-like signum.

###### Description.

**Figure 2.**
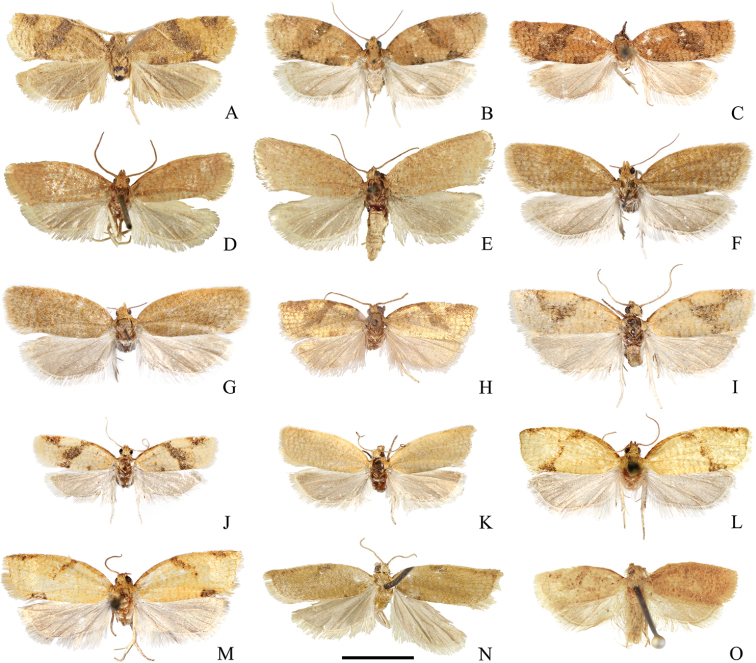
Adults of *Clepsis neglectana* species group **A–C***C. neglectana*: **A** lectotype of *Tortrix neglectana***B** male, Southern Germany **C** Female, Germany **D–G***C. striolana*: **D** lectotype of *T. striolana***E** paralectotype of *T. striolana***F** male, Austria, South Tyrol **G** female, Austria, South Tyrol **H–K***C. trivia*: **H** holotype of *T. trivia***I–J** males, Crete **K** female, Crete **L–M***C. acclivana*: **L** lectotype of *Cacoecia acclivana***M** paralectotype of *C. acclivana***N***T. severana* holotype **O**Cacoecia unifasciana var. semiana holotype. Scale bar: 5 mm, all to scale.

**Figure 3.**
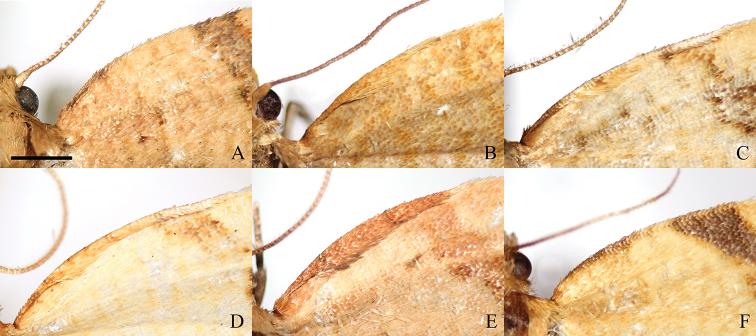
Costal folds of males of *Clepsis* spp. **A***C. neglectana*, Germany **B***C. striolana*, Austria **C***C. trivia*, Crete **D***C. acclivana*, lectotype of *Cacoecia acclivana***E***C. consimilana*, neotype of *Tortrix consimilana***F***C. eatoniana*, Spain. Scale bar: 1 mm, all to scale.

**Figure 4.**
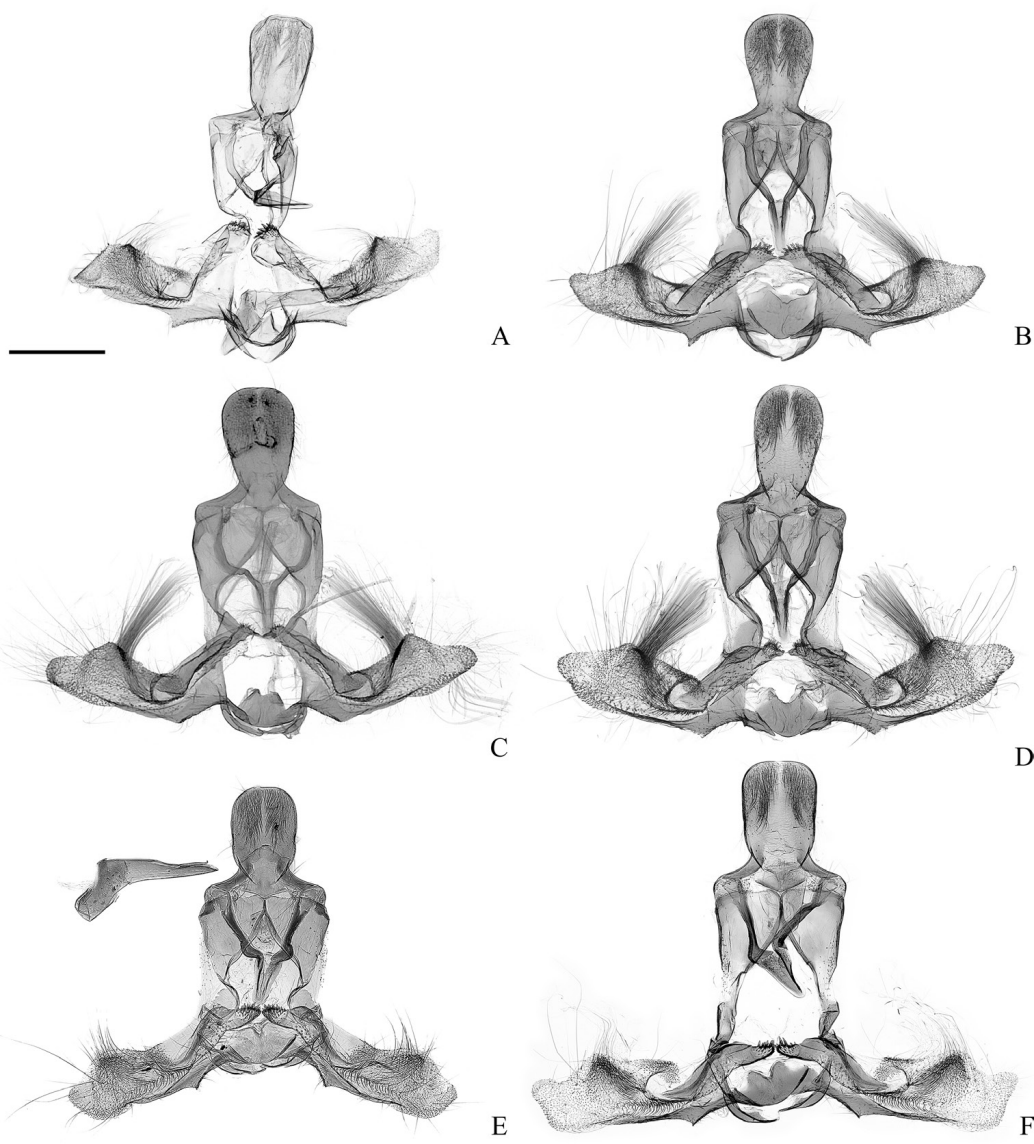
Male genitalia of the *Clepsis neglectana* species group (some without phallus) **A, B***C. neglectana*: **A***Tortrix neglectana* lectotype **B***C. neglectana*, Southern Germany **C, D***C. striolana*: **C***T. striolana* lectotype **D***C. striolana*, Austria, North Tyrol **E, F***C. trivia*: **E***T. trivia* holotype **F***C. trivia*, Crete. Scale bar: 500 μm, all to scale.

**Figure 5.**
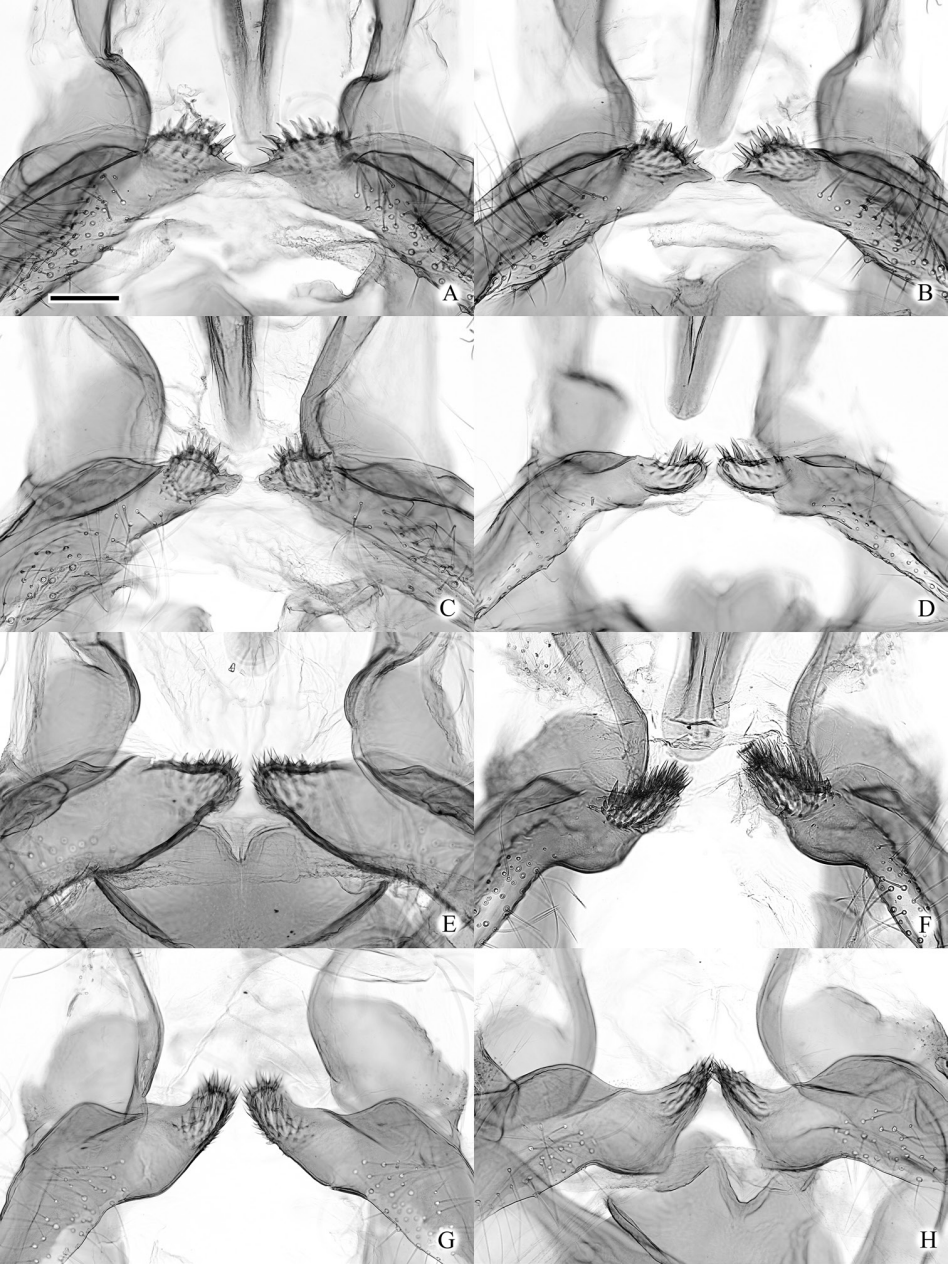
Labides of *Clepsis* spp. **A, B***C. neglectana*: **A** Southern Germany **B** Finland **C***C. striolana*, Austria, North Tyrol **D***C. trivia*, Crete **E***C. acclivana*, paralectotype of *Cacoecia acclivana***F, G***C. consimilana*: **F** Germany, neotype of *Tortrix consimilana***G** Italy **H***C. eatoniana*, Spain. Scale bar: 100 μm, all to scale.

**Figure 6.**
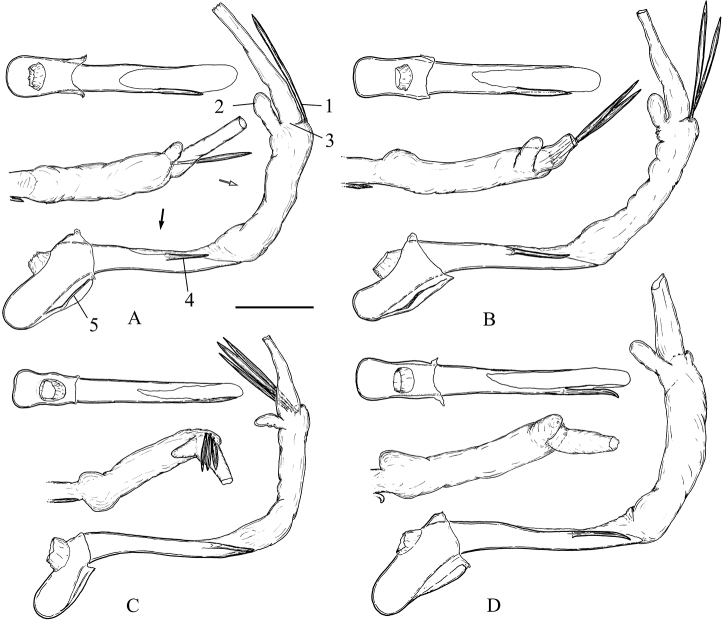
Phalli with vesicae everted of the *Clepsis neglectana* species group **A***C. neglectana*, Germany **B***C. striolana*, Austria **C***C. trivia*, Crete **D***C. acclivana*, paralectotype of *Cacoecia acclivana*. The phallus of each species is shown in three aspects from top to bottom: sclerotised phallic tube (without vesica) in dorsal view (viewpoint marked with black arrow), vesica in dorsal view (viewpoint marked with white arrow) and whole phallus in left view. 1 cornutus, 2 diverticulum, 3 location of gonopore, 4 phallic process, 5 caulis. Scale bar: 250 μm.

**Figure 7.**
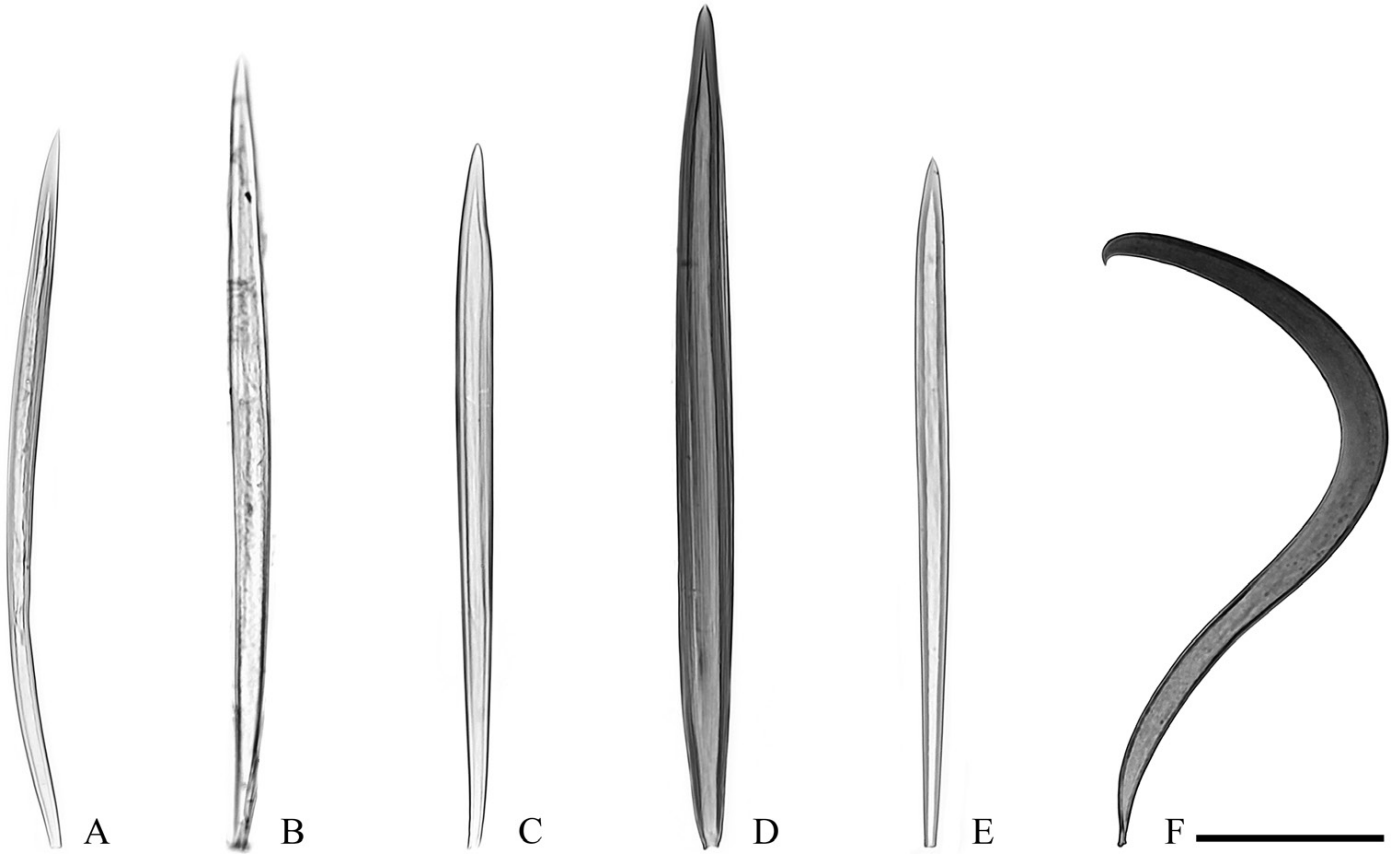
Specialised male setae in *Clepsis* spp. **A–E** cornuti: **A***C. neglectana*, Finland **B***C. striolana*, Austria **C***C. trivia*, Crete **D***C. consimilana*, neotype of *Tortrix consimilana***E***C. eatoniana*, Spain **F** modified large seta from the medial surface of valva of *C. consimilana*, Germany. Scale bar: 100 μm, all to scale.

**Figure 8.**
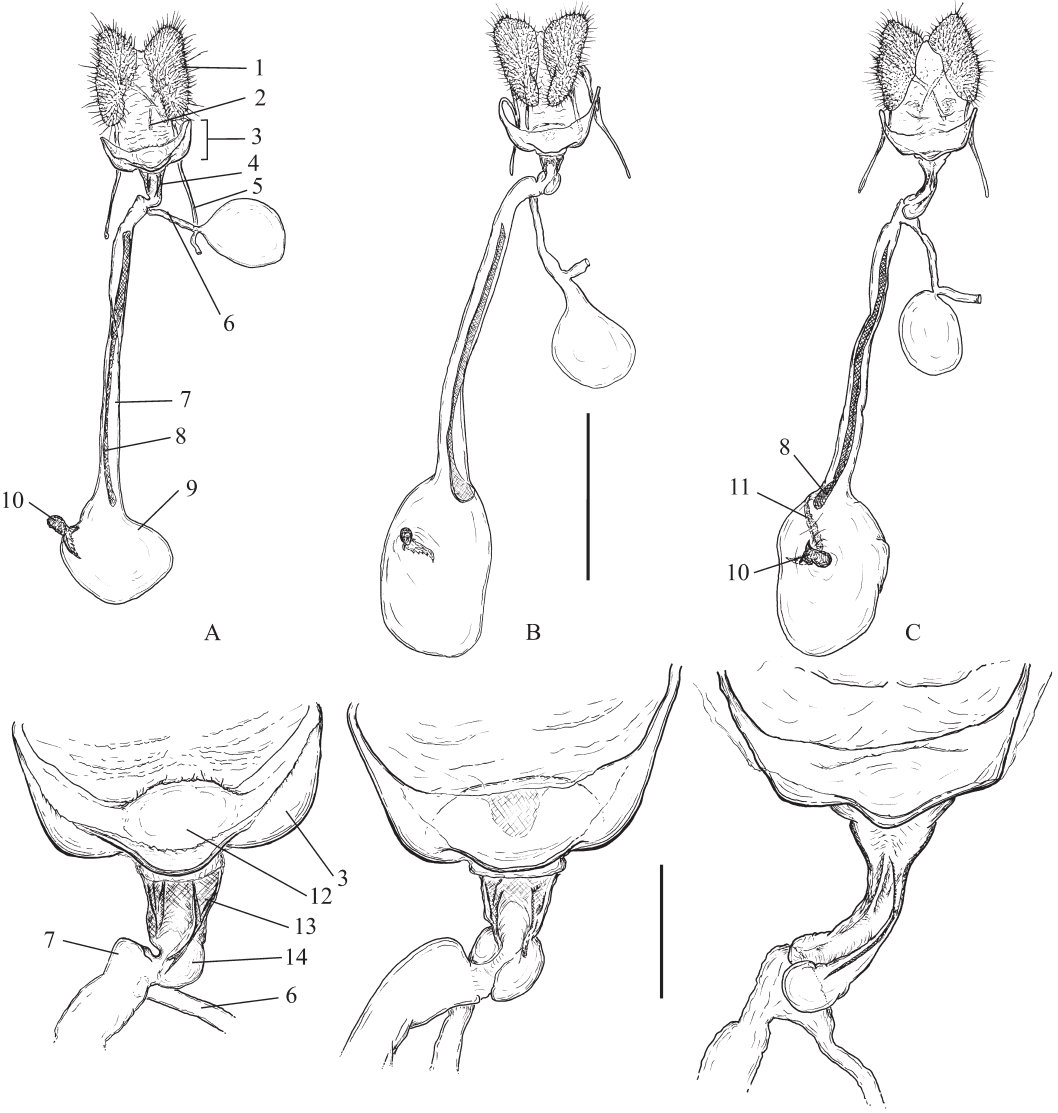
Female genitalia of the *Clepsis neglectana* species group **A***C. neglectana*, Finland **B***C. striolana*, Austria **C***C. trivia*, Crete. The top row shows the whole genitalia, the bottom row shows the sterigma and colliculum enlarged. 1 papilla analis, 2 apophysis posterior, 3 sterigma, 4 colliculum, 5 apophysis anterior, 6 ductus seminalis, 7 ductus bursae, 8 cestum, 9 corpus bursae, 10 falcate signum, 11 plate shaped signum, 12 ostium, 13 sclerotised plicae, 14 protrusion. Scale bars: 1 mm (top row), 250 μm (bottom row).

**Figure 9.**
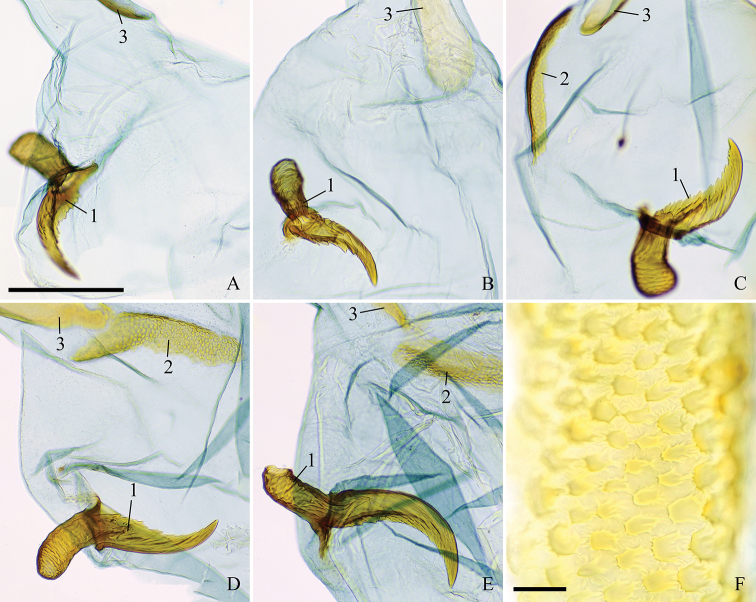
Signa of bursa copulatrix in *Clepsis* spp. **A***C. neglectana***B***C. striolana***C***C. trivia***D***C. consimilana***E***C. eatoniana***F** A detail of plate shaped signum of *C. consimilana* consisting of sclerotised papillae. 1 falcate signum, 2 plate shaped signum, 3 cestum. Scale bars: 250 μm (**A–E**, all to scale), 25 μm (**F**).

Adult (Fig. 2A–C). Sexual dimorphism not detected. Head. Vertex, frons, palps and antennae monochrome, covered with ochreous scales. Sensilla trichodea (often referred to as “cilia”) on antennae denser and longer in males. Thorax dorsally, legs and tegula ochreous, thorax ventrally creamy. Forewing length in males 6.3–7.4 mm (mean 6.9, *N* = 10), in females 6.5–8.2 mm (mean 7.3, *N* = 4). Forewing elongated, with costa convex basally and slightly concave apically, costal fold rudimentary (Fig. 3A). Upperside background ochreous to ferruginous with darker transverse or reticulate pattern. Markings brown to grey brown: basal blotch usually ill-defined, expressed mainly at costa and dorsum; median fascia widened at the middle. Subapical blotch triangular, ill-defined, sometimes connected with the median fascia. Cilia concolourous or paler than background. Underside pale grey-brown, sometimes with ill-defined reticulate pattern and creamy longitudinal blotch in the distal half of costa. Hindwing grey on both sides with underside paler, cilia whitish with grey line. Abdomen grey. Male genitalia (Fig. 4A, B). Uncus ovoid, widening apically, rounded, gnathos relatively large, socius membranous. Valvae pointed dorsolaterally when mounted on slide. Costal sclerite of valva relatively narrow, with short elliptic labis covered with small acanthae and extended into triangular pointed medial process (Fig. 5A, B). Apical part of sacculus ca. 1.4× longer than basal part, both forming angle of 145–155°, saccular process pointed. Membranous part of valva with protuberance bearing tuft of firmly attached, relatively sparse scales and setae; its terminal part with concave dorsal and convex ventral margin, brachiola ill-defined, pointed dorsolaterally. Posterior part of phallus slightly bent dorsally, with lateral process as long as 0.23× distance between anterior opening and tip of phallus, straight, in single specimen apically bent dorsally. Anterior and posterior part of phallus form angle of 130–140°. Caulis large, L-shaped, parallel to coecum. Vesica bent at ca. 110° dorsally, with small basal widening and terminal diverticulum mediodorsally, slightly pointed to right (Fig. 6A). One long, slightly curved deciduous cornutus attached ventroterminally adjacent to gonopore (Fig. 7A); single specimen with two cornuti. Female genitalia (Fig. 8A) with papillae anales not modified. Apophyses anteriores 1.3× longer than apophyses posteriores. Sterigma widened caudad, with shallow lateral sclerotised pockets cephalad and large excavation on the dorsal wall. Colliculum short, with length 0.14× length of ductus bursae, straight, funnel-shaped, with plicate longitudinal sclerotisation and lateral protrusion at right cranial end consisting of colourless thick cuticle. Ductus bursae long and narrow, emerging at left to cuticular protrusion, with cestum extending along cranial 0.9× of its length and expanding for short distance on corpus bursae. Ductus seminalis inserted dorsally at caudal end of ductus bursae. Corpus bursae ovoid, with large falcate signum with capitulum (Fig. 9A).

Preimaginal stages are unknown.

###### Molecular data

(Fig. 16). BIN: BOLD:AAM0282. DNA barcodes identical (*N* = 2). The minimum distance to the nearest BIN-sharing neighbour, *C. striolana*, is 1.53%.

###### Distribution

(Fig. 17). Central and Northern Europe. Previous records from other parts of the Palaearctic (e.g., Wang et al. 2003) need reconsideration.

###### Ecology.

Moths were collected in July and the beginning of August. The larval host plant is stated as *Fragaria* (Razowski 2002) but due to repeated misidentifications this record as well as published habitats need verification.

###### Remarks.

This is the oldest described taxon from the group. Many other taxa were subsequently synonymised, but at least three of them are *species bona* and three others are *incertae sedis* (see below).

##### Clepsis striolana

Taxon classificationAnimaliaLepidopteraTortricidae

(Ragonot, 1879) stat. rev.

BE460CED-33FF-58A3-85F7-946C8220C25C

Tortrix striolana Ragonot, 1879: 132 (Switzerland)

###### Material examined.

Lectotype ♂ by designation of Razowski (1979), pinned, with 5 labels: “*Tortrix* / *striolana* Rag. / Bull. Soc. ent. Fr., / 1879, p. 132.” [handwritten] “striolatana [sic] / Rag. Helv.” [handwritten] “Type” [printed red] “1901 / coll. E. L. Ragonot / Muséum Paris” “Zool. Mus. Berlin / Genit. – Unters. / Nr. 287.” [printed]; male genitalia on a slide with two labels: “287. / Clepsis / striolana / Ragonot / Type.” “287. Clepsis / striolana Rag. / Helv. / Bull. Soc. Ent. / Fr. 1879 p. 132 / Type.” [both handwritten, with red border].

SWITZERLAND • 1 ♂; E. L. Ragonot leg.; GS 287; MNHN.

Paralectotype ♀ pinned, with three labels: “Striolatana [sic] / Rag. Helv.” [handwritten] “1901 / coll. E. L. Ragonot / Muséum Paris” [printed] ‘Allotype’ [handwritten red].

SWITZERLAND • 1 ♀; E. L. Ragonot leg.; MNHN.

Other material: AUSTRIA • 2 ♂♂; North Tyrol, Stanzach, Blockau; alt. 920 m; 5 Jul. 1989; P. Huemer leg.; GS TOR 6; TLMF • 3 ♂♂, 1 ♀; same collection data; 16 Jul. 1989; P. Huemer leg.; GS 1/11.10.2017, 2/11.10.2017; TLMF • 6 ♂♂, 2 ♀♀; same collection data; 26 Jul. 1989; P. Huemer leg.; GS 3/11.10.2017; TLMF • ITALY • 3 ♂♂; Südtirol, Prad, Praderfeld; alt. 900 m; 9 Jul. 1991; P. Huemer leg.; TLMF • 1 ♂; same collection data; 20. Jun. 1998; T. Mayr leg.; TLMF; • 1 ♂, 1 ♀; prov. Torino, Val Chisone, Fenestrelle; alt. 1150 m; Aug. 1928; G. Della-Beffa leg.; NHMW.

###### Diagnosis.

This is the only species of the *C. neglectana* group with a uniform wing pattern in both sexes. Unlike the males of *C. neglectana*, the costal fold is developed. The male genitalia are very similar to *C. neglectana* but the setal tuft of the valva is better developed, the uncus is slightly longer, the sacculus is more angled, the vesica bears two cornuti instead of one, and the shape of the labis is different. The female genitalia are also similar to *C. neglectana*, although some differences in the colliculum (the transparent cuticular protrusions) are present. The colliculum is much shorter and straighter in *C. striolana* than in *C. trivia*.

###### Description.

Adult (Fig. 2D–G). Sexual dimorphism not detected. Head. Vertex, frons, palps and antennae monochrome, covered with ochreous scales. Sensilla trichodea on antennae denser and longer in males. Thorax dorsally, legs and tegula ochreous, thorax ventrally creamy. Forewing with length 8.0–8.2 mm (mean 8.1, *N* = 3), relatively wide, with costa convex basally and straight apically, costal fold small, extending from base to 0.4× length of costal margin (Fig. 3B). Upperside background ochreous with fulvous reticulate pattern, underside pale grey-brown with creamy margins, cilia pale ochreous. Hindwing upperside pale grey, underside whitish, cilia whitish with grey line. Abdomen grey. Male genitalia (Fig. 4C, D). Uncus ovoid, widening dorsally, rounded, gnathos relatively large, socius membranous. Valvae pointed dorsolaterally when mounted on slide. Costal sclerite of valva relatively narrow, with short round labis covered with small acanthae and extended into pointed medial process (Fig. 5C). Apical part of sacculus ca. 1.4× longer than basal part, both forming an angle of 135–140°, saccular process nearly right angled. Membranous part of valva with protuberance bearing compact tuft of dense, firmly attached scales and setae; its terminal part with concave dorsal and convex ventral margin, brachiola large, pointed laterally. Posterior part of phallus straight, with lateral process as long as 0.28× distance between anterior opening and tip of phallus, apically bent dorsally. Anterior and posterior part of phallus form angle of 135–145°. Caulis large, Z-shaped, parallel to coecum. Vesica bent at ca. 130° dorsally, with small basal widening and terminal diverticulum dorsolaterally, pointed to right (Fig. 6B). Two long, slightly curved deciduous cornuti attached ventroterminally adjacent to gonopore (Fig. 7B). Female genitalia (Fig. 8B) with papillae anales not modified. Apophyses anteriores 1.7× longer than apophyses posteriores. Sterigma widened caudad, with shallow lateral sclerotised pockets cephalad and large excavation on the dorsal wall. Colliculum short, with length 0.15× length of ductus bursae, straight, funnel-shaped, with plicate longitudinal sclerotisation, larger right and smaller left lateral protrusions at cranial end consisting of colourless thick cuticle. Ductus bursae long and narrow, emerging at left between cuticular protrusions, with cestum extending along cranial 0.9× of its length and expanding for short distance on corpus bursae. Ductus seminalis inserted dorsally at caudal end of ductus bursae. Corpus bursae ovoid, with large falcate signum with capitulum (Fig. 9B).

Preimaginal stages are unknown.

###### Molecular data

(Fig. 16). BIN: BOLD:AAM0282. The intraspecific average and maximum distances of the barcode region is unknown (*N* = 1). The minimum distance to the nearest BIN-sharing neighbour, *C. neglectana*, is 1.53%.

###### Distribution

(Fig. 17). This species seems to be limited to the Alps: Switzerland, Austria and Italy.

###### Ecology.

Moths were collected from late June to August on sparse scree along alpine rivers. The larval host plant is unknown.

###### Remarks.

The genitalia of this species resemble those of *C. neglectana*, only a few details in certain structures are different. The genetic distance (though small) and wing pattern both support the existence of two separate taxa.

##### Clepsis trivia

Taxon classificationAnimaliaLepidopteraTortricidae

(Meyrick, 1913), stat rev.

4840A373-38BE-577A-B380-6AA9E023B9AA

Tortrix trivia Meyrick, 1913: 297 (Tunisia)

###### Material examined.

Holotype ♂, pinned, with 6 labels: “Tunis / 27.5 / Coll. O. Leonhard” “T / 29” “Tortrix / trivia Meyr. / type” “Holotypus” [red label] “DEI Müncheberg / Lep-00335” [green label] “Gen.-Präp. / 3145 / präp. Karisch, 2014”.

TUNISIA • 1 ♂; 27 May; Leonhard leg.; GS 3145; SDEI Lep-00335.

Other material: GREECE • 1 ♀; Crete, Lassithi region, Agios Joannis; alt.250 m; 28 Apr. 2003; W. Ruckdeschel leg.; GS 1/12.10.2017; TLMF • 1 ♀; same collection data; 1 May 2003; GS 2/12.10.2017; TLMF • 2 ♂♂; Crete, Lassithi region, Koutsounari; alt. 100 m; 1 May 2003; W. Ruckdeschel leg.; GS 1/13.10.2017, 2/13.10.2017; TLMF • 1 ♀; Crete, Lassithi region, Achlia at Koutsouras; alt. 30 m; 5 Nov. 2004; W. Ruckdeschel leg.; GS 3/13.10.2017; TLMF • 2 ♂♂, 1 ♀; same collection data; 7 Nov. 2004; GS 4/13.10.2017, 5/13.10.2017, 6/13.10.2017; TLMF • 1 ♀; same collection data; 9 Apr. 2008; GS 1/14.10.2017; TLMF • 3 ♂♂; same collection data; 12 Apr. 2008; GS 1/15.10.2017, 2/15.10.2017, 3/15.10.2017; TLMF.

###### Diagnosis.

The species is most similar to *C. acclivana*. Externally the males differ in their wing markings, which are better defined in *C. acclivana*. The wings in *C. trivia* are also more elongated. The gnathos of *C. trivia* has angled arms, the sacculus is straighter, the brachiola is displaced dorsally, the phallic process has a different orientation but the vesica is very similar to *C. acclivana*. The wing pattern of *C. trivia* females is similar to those of both the *C. consimilana* and *C. neglectana* groups, but is *C. trivia* is paler. *Clepsis trivia* has the longest colliculum among the known females of the *C. neglectana* group.

###### Description.

Adult. Sexual dimorphism prominent. Male (Fig. 2H–J). Head. Vertex pale fulvous, frons, palps and antennae with ochreous scales. Antennae with numerous sensilla trichodea as long as width of flagellomeres. Thorax dorsally fulvous, ventrally creamy, legs pale brown, tegula creamy with fulvous anterior part. Forewing relatively elongate, with length 6.1–7.9 mm (mean 7.3, *N* = 7). Costa convex basally and straight apically, with small costal fold extending from base to 0.4–0.5× length of costal margin (Fig. 3C). Background pale yellowish with ill-defined reticulate pattern. Basal blotch ill-defined, consisting of small transverse fulvous markings. Median fascia brown or fulvous, widened at middle. Subapical blotch triangular, ill-defined, connected with median fascia. Underside pale grey-brown with creamy area in distal half of costa. Cilia concolourous with background. Hindwing upperside pale grey, underside whitish, cilia white. Abdomen pale grey. Male genitalia (Fig. 4E, F). Uncus wide, more or less round apically, with parallel lateral margins, gnathos with relatively large median part and angled arms, socius membranous. Valvae pointed laterally or slightly dorsolaterally when mounted on slide. Costal sclerite wide, with elongated labis covered with large acanthae (Fig. 5D). Apical part of sacculus 1.6× longer than basal part, both forming an angle of 150–160°, saccular process pointed, relatively small. Membranous part of valva with protuberance lacking tuft of setae but has deciduous scales, terminal part with small dorsal and large ventral curvature, brachiola prominent, pointed dorsally. Posterior part of phallus slightly sinuate, with lateral process 0.24× distance between anterior opening and tip of phallus, weakly curved dorsad or rarely parallel to tip. Anterior and posterior part of phallus form angle of 130°. Caulis small, diverging from coecum. Vesica bent at 110–130° dorsad, with basal widening and terminal diverticulum dorsally, slightly pointed to right (Fig. 6C). Three ventroapically located deciduous cornuti adjacent to gonopore (Fig. 7C). Female more unicolourous than male (Fig. 2K). Head as in male but sensilla trichodea less numerous and shorter. Thorax as in male but tegula fulvous. Forewing length 6.6–7.9 mm (mean 7.3, *N* = 5). Costa convex basally and slightly concave apically. Upperside background pale yellowish with pale fulvous reticulate pattern, without markings, underside pale grey-brown with creamy area in distal half of costa, cilia concolourous with upperside. Hindwings upperside pale grey, underside and cilia whitish. Abdomen grey. Female genitalia (Fig. 8C). Papillae anales not modified. Apophyses anteriores slightly longer (1.1×) than apophyses posteriores. Sterigma widened caudad, with small shallow lateral pockets cephalad and large excavation on dorsal wall, lamella antevaginalis narrow. Colliculum asymmetrical, with length 0.2× length of ductus bursae, funnel-shaped, bent to left, with plicate longitudinal sclerotisation, large elongated protrusion at right and a small one at left both consisting of colourless thick cuticle. Ductus bursae long and narrow, emerging dorsally of cuticular protrusions. Cestum expanding for short distance on corpus bursae and extending along cranial part of ductus bursae for 0.9× of its length. Ductus seminalis inserted dorsolaterally at caudal end of ductus bursae. Corpus bursae ovoid, with large falcate signum with capitulum and flat signum located near end of cestum consisting of sclerotised papillae (Fig. 9C).

###### Molecular data

(Fig. 16). BIN: BOLD:ACT3810. The intraspecific average distance of the barcode region is 0.08%, the maximum distance 0.16% (p-dist) (*N* = 4). The minimum distance to the nearest neighbour, *C. eatoniana*, is 3.34%.

###### Distribution

(Fig. 17). Known from Tunisia (type locality) and Crete.

###### Ecology.

The moths fly in the middle of April to late May and in early November, which may indicate two generations per year.

###### Remarks.

Meyrick (1913) described *C. trivia* from a single male. The name remained valid until Karisch and Blackstein (2014) dissected the holotype (by monotypy) and synonymised it with *C. neglectana*. Despite of proposed synonymy, they explicitly stated that *C. acclivana* and *C. trivia* are very similar to each other but differ from *C. neglectana*. We find very little support for synonymy between *C. neglectana*, *C. trivia*, and *C. acclivana*. The holotype of *C. trivia* has very similar wing pattern to the males collected in Crete, and the male genitalia (apart from the unstudied vesica of the holotype) appear identical, therefore the Cretan population can be assigned to *C. trivia* despite lacking barcode data for the holotype. There is a considerable DNA barcode gap between *C. trivia* (from Crete) and *C. neglectana* (from Europe) which also supports existence of two taxa.

##### Clepsis acclivana

Taxon classificationAnimaliaLepidopteraTortricidae

(Zerny, 1933) stat. rev.

2C69A24D-3996-5AF3-98D5-2A8EF7FE9022

Cacoecia acclivana Zerny, 1933:108, pl. 1, fig. 11 (Lebanon)

###### Material examined.

Lectotype ♂ by designation of Razowski (1979), pinned, with 6 labels: “Nord-Libanon / Becharré, 1400 m / 21.–28.vi.[19]31. Zerny” “Cacoecia / acclivana / Zerny Type!” [handwritten] “Cacoecia” ♂ / acclivana Zerny / N. Obraztsov det. 1965 / prep. No. V. 49” [handwritten and printed] “Lectotype” [green label] “Nat. hist. Mus. / Wien / Gen. Praep. / MV 2533” [blue label] “NHMW / Type fot / 2013”; male genitalia on a slide with two labels: “Cacoecia” / acclivana / Zerny / Nord-Libanon / Becharré / 1400 m 21.–28.vi / 1931 Zerny” “♂ / V. 49 / Mus. Vind. 2533 / Lectotypus” [both handwritten].

LEBANON • 1 ♂; Bsharri; alt. 1400 m; 21–28 Jun. 1931; Zerny leg.; GS V. 49; NHMW 2533.

Paralectotype ♂, pinned, with four labels: “Nord-Libanon / Becharré, 1400 m / 11.–20.vi.[19]31. Zerny” “Lectoparatype” [green label] “Cacoecia / acclivana / Zerny Type!” [handwritten] “NHMW / Gen. Prep. ♂ / No. 1/15.2.2018”; male genitalia on a slide with two labels: “Paralectotype / *Cacoecia acclivana* / Zerny, 1933 / Nord-Libanon, Becharré, 1400 / m, 11.–20.vi.[19]31, Zerny” [red label] “NHMW / Gen. prep. / ♂ / No. 1/15.2.2018 / B. Zlatkov 2018 Euparal”.

LEBANON • 1 ♂; Bsharri; alt. 1400 m; 11–20 Jun. 1931; Zerny leg.; GS 1/15.2.2018; NHMW.

###### Diagnosis.

*Clepsis acclivana* is most similar to *C. trivia* but the forewings are paler and wider with more distinct markings, the uncus is narrower, the median part of the gnathos is smaller and its arms are not angled, the sacculus is more curved, the labis is more massive and with shorter acanthae, and the apex of the phallic process is curved ventrolaterally instead of dorsally.

###### Description.

**Figure 10.**
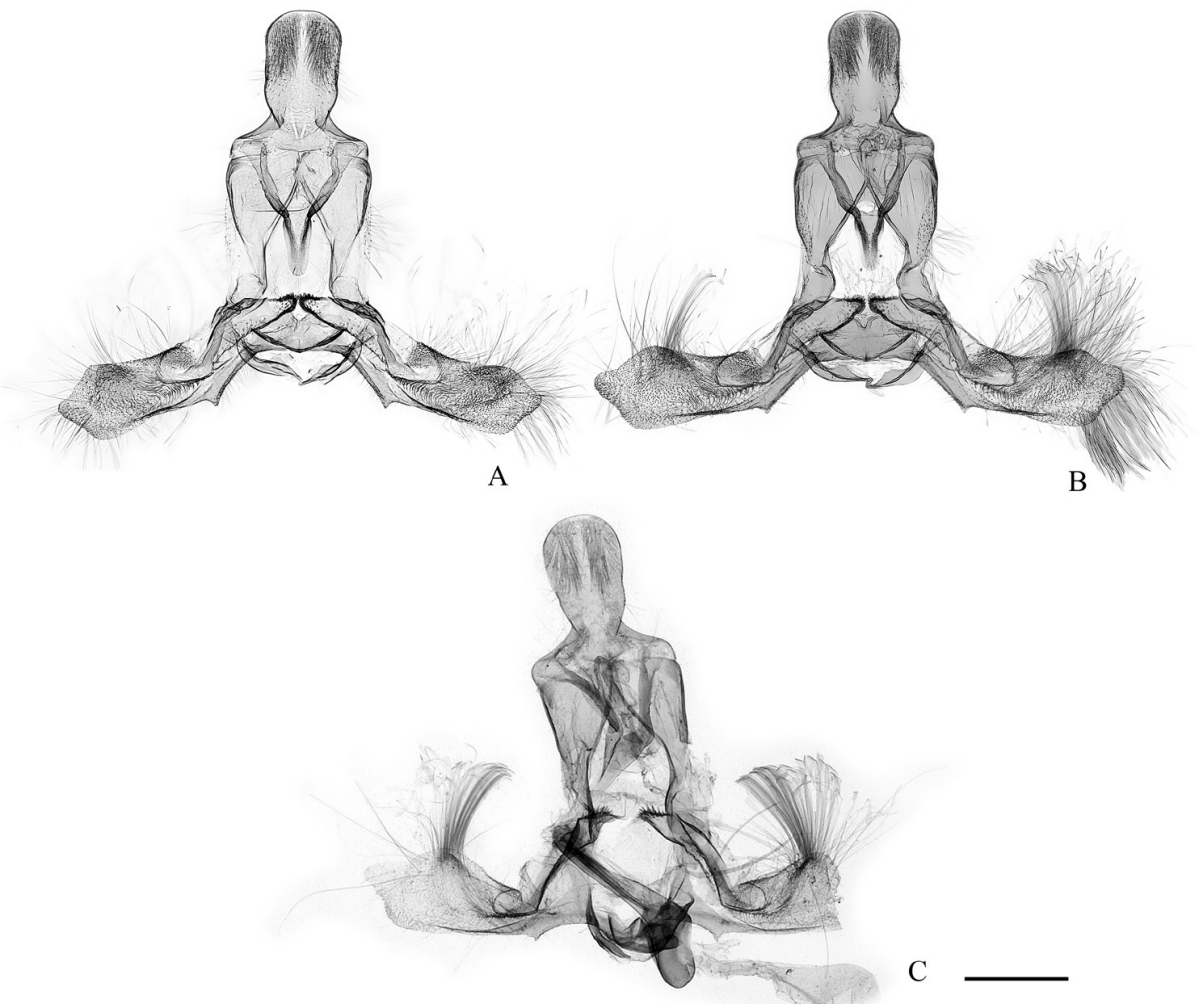
Male genitalia of the *Clepsis neglectana* species group (some without phallus) **A–B***C. acclivana*: **A***Cacoecia acclivana* lectotype **B***C. acclivana* paralectotype, right valva intentionally not cleaned from scales **C***Clepsis semiana*, holotype of Cacoecia unifasciana var. semiana. Scale bar: 500 μm, all to scale.

Adult. Sexual dimorphism unknown. Male (Fig. 2L, M). Head. Vertex pale fulvous, frons, palps and antennae with ochreous scales. Antennae with numerous sensilla trichodea as long as width of flagellomeres. Thorax dorsally pale fulvous, ventrally creamy, legs pale brown, tegula pale fulvous. Forewing with length of 7.8 mm (in both specimens), costa basally convex, apically straight, with costal fold extending from base to 0.4× length of costal margin (Fig. 3D). Upperside background pale yellowish with fulvous reticulate pattern. Markings ill-defined, consisting of brown and ferruginous scales: basal blotch faint, with remnants only at costa as dark line; median fascia narrow, more prominent at dorsum; subapical blotch reduced, dash-like. Cilia concolourous with background. Underside pale grey-brown, costal and terminal areas creamy with some reticulate pattern. Hindwings upperside monochrome pale grey, cilia concolourous, underside whitish. Abdomen pale grey. Male genitalia (Fig. 10A, B). Uncus round apically, with parallel lateral margins, gnathos relatively small, socius membranous. Valvae pointed laterally or ventrolaterally when mounted on slide. Costal sclerite of valva very wide, with short wide labis covered with small acanthae (Fig. 5E). Basal and apical parts of sacculus with equal length forming angle of ca. 140°, saccular process almost right-angled, relatively large. Membranous part of valva with protuberance devoid of tuft of setae but has deciduous scales; its terminal part with nearly symmetrical dorsal and ventral curvature, brachiola prominent, pointed laterally. Posterior part of phallus slightly sinuate, with lateral process as long as 0.29× distance between anterior opening and tip of phallus, apically bent ventrolaterally. Anterior and posterior part of phallus form angle of 130°. Caulis large, diverging widely from coecum. Vesica bent at ca. 110° dorsad, with basal widening and terminal diverticulum dorsally, slightly pointed to right. Three sockets of ventroapically located deciduous cornuti adjacent to gonopore are detectable (Fig. 6D).

Female unknown.

Preimaginal stages unknown.

###### Molecular data.

Unknown.

###### Distribution.

Known from the type locality only: Lebanon (Fig. 17).

###### Ecology.

Not known.

###### Remarks.

Comparison of the wing pattern and genitalia of the lectotype and paralectotype of *C. acclivana* with the species considered above confirmed the assumption that it is a distinct species. The barcoding distance to the other species was not studied because of lack of fresh material, but the great similarity in all morphological characters of the two specimens convinced us that they can represent a distinct species, therefore we resurrect the initial status of the taxon *acclivana* synonymised by Razowski (1979) with *C. neglectana*.

###### Taxa incertae sedis.

Without further morphological or genetic support the following species cannot be interpreted with certainty, but the synonymy with *C. neglectana*, *C. acclivana* or *C. trivia* does not seem justified for now. They demonstrate some morphological gap; at least the differences between them are not smaller than the differences with the above mentioned species of the group. Additional material and genetic study is necessary to solve their real status.

##### Clepsis severana

Taxon classificationAnimaliaLepidopteraTortricidae

(Kennel, 1901) stat. rev.

D1A8D990-8BA1-56C8-8F6E-89E06B71EFCC

Tortrix severana Kennel, 1901: 227 (Algeria)

###### Remarks.

The species was synonymised with *C. neglectana* by Razowski (1979) based on examination of the genitalia of the female holotype (Fig. 2N). The slide subsequently was lost and we were unable to re-examine the genitalia. Regarding the type locality (Algeria) it seems unlikely that this taxon is conspecific with *C. neglectana*.

##### Clepsis semiana

Taxon classificationAnimaliaLepidopteraTortricidae

(Chrétien, 1915) stat. nov.

E3DC50DF-B8FF-5694-B3CC-9DDC78D286B1

Cacoecia unifasciana var. semiana Chrétien, 1915: 296 (Tunisia)

###### Material examined.

Holotype ♂: pinned, with 4 labels: “Cacoecia / unifasciana / v. semiana” [handwritten] “Type” [red, printed] “8.6” [handwritten] “Holotype / *Clepsis semiana* / (Chrétien, 1915) / Zlatkov & Huemer, 2019 des.” [red, printed].

TUNISIA • 1 ♂; MNHN.

###### Remarks.

The male genitalia of *C. semiana* (Fig. 10C) are very similar to those of *C. striolana*. However, there is a clear difference in the wing pattern. Though the lectotype of C. unifasciana var. semiana is poorly preserved, some poorly defined markings can be detected (Fig. 2O). The physical distance between the populations of this taxon and *C. striolana* is considerable and it is very unlikely that var. semiana is conspecific. The synonymy between *C. semiana* and *C. consimilana* proposed by Obraztsov (1955) is not justified. Apparently, it is rooted in the initial incorrect assignment of var. striolana to *Cacoecia unifasciana*, but the latter turned out to be a junior synonym of Clepsis consimilana (see below) . The taxon var. semiana was mechanically moved to the synonymic list of *C. consimilana* without studying the genitalia of the type specimen.

#### The *Clepsis consimilana* species group

This species group is characterised by the presence of large modified setae on the median surface of the valvae (Fig. 1).

##### Clepsis consimilana

Taxon classificationAnimaliaLepidopteraTortricidae

(Hübner, 1817)

C929F103-54A0-5685-B877-430BB5355771

[Tortrix] consimilana Hübner, 1817: pl. 38, fig. 239 (Europe).
Tortrix unifasciana Duponchel, 1842: 135, pl.61, fig. 6 (France).
Tortrix unifasciana f. cinnamomeana Dufrane, 1957: 6 (Belgium).
Tortrix fallaciana Fuchs, 1903: 4 (Sicily).
Tortrix unifasciana f. fuscana Dufrane, 1957: 6 (Belgium).
Tortrix unifasciana f. obliterana Dufrane, 1957: 6 (Belgium).
Paramesia peregrinana Stephens, 1852: 90 (Britain), unavailable, published in synonymy with Lozotaenia obliquana Humphreys and Westwood 1845.
Tortrix productana Zeller, 1847: 660 (Sicily).
obliterana Herrich-Schäffer, 1847 (uninominal): pl. 9, fig. 60.
Tortrix (Lozotaenia) obliterana Herrich-Schäffer, 1851: 164 (Italy).
Siclobola placida Diakonoff, 1948: 25 (Madagascar).
non Tortrix eatoniana Ragonot, 1881
non Cacoecia unifasciana var. semiana Chrétien, 1915
non Cacoecia acclivana Zerny, 1933

###### Material examined.

Neotype ♂ (here designated): pinned, with 5 labels: “Gehegs / Dresden / 18.vi.1922 / E. Möbius” “Coll. E. Möbius / Ankauf 1946” “Neotype / *Tortrix consimilana* / Hübner, 1817 / Zlatkov & Huemer, 2019 des.” [red printed] “Museum für Tierkunde Dresden / Gen. prep. / ♂ / No. 2/30.11.2018” “*Clepsis consimilana* / (Hübner, 1817) / Zlatkov & Huemer, 2019 det.”; genitalia on a slide with two labels: “Neotype / *Tortrix consimilana* / Hübner, 1817 / [Germany] Dresden, Gehegs / 18.vi.1922, E. Möbius / Zlatkov & Huemer, 2019 des.” [red printed] “Museum für Tierkunde Dresden / Gen. Prep. / ♂ / No. 2/30.11.2018 / B. Zlatkov 2018 Euparal”.

GERMANY • 1 ♂; Dresden, Gehegs; 18 Jun. 1922; E. Möbius leg.; GS 2/30.11.2018; MTD.

Lectotype ♂ of *Tortrix unifasciana* (here designated): pinned, with 5 labels: “unifasciana” [handwritten] “Duponch[el]” [round, handwritten] “713” [round handwritten] “Type” [red printed] “Chr. Gibeaux dét. / prép. génit. n° 4231 ♂ / unifasciana / Dup.” [printed and handwritten]; male genitalia on a slide with two labels: “Tortrix / unifasciana / Dup., 1842 / Type” [handwritten with red border] “Chr. Gibeaux dét. / prép. génit. n° 4231 ♂ / Clepsis / consimilana Hb. / 30.x.91 Euparal M. P.” [printed and handwritten].

FRANCE • 1 ♂; GS 4231; MNHN.

Holotype ♂ of *Siclobola placida*: pinned, with four labels: “Tananarive” “Type: ♂ / Siclobola / placida / A. Diakonoff 1946” [printed and handwritten] “Holotype” [printed red] “Siclobola / unifasciana / placida / 1959 Diak. / det. A. Diakonoff” [printed and handwritten]; genitalia on glass slide pinned to the holotype, with one label: “536.D.”

MADAGASCAR • 1 ♂; Antananarivo; MNHN 536.D.

Other material: GERMANY • 1 ♂; Dresden, Gehegs; 24 Jun. 1921; E. Möbius leg.; GS 1/30.11.2018; MTD • 1♂; Dresden, Lössaltz; 28 Jun. 1935; E. Möbius leg.; GS 2/3.12.2018; MTD • 1 ♂; same collection data; 3 Aug. 1935; GS 1/3.12.2018; MTD • 1 ♀; Fritzsche leg.; GS 1/4.12.2018; MTD • 1 ♂; Sylt; GS 2/5.11.2018; ZSM • 2 ♂♂, 2 ♀♀; Baden Württemberg, Marbach am Neckar; 29 Jun. 1957; L. Süssner leg.; TLMF • 1 ♂; Bayern, München, Hochmutting; 19 Aug. 1988; H. Kolbeck leg.; TLMF • 1 ♀; Kehlheim; 12 Jun. 1997; H. Kolbeck leg.; TLMF • ITALY • 1 ♂; prov. Verona, Monte; alt. 300 m; 20 Aug. 1994; Franz leg.; GS 1/16.10.2017; TLMF • 1 ♂, same collection data; 7 Jun. 1991; K. Burmann et al. leg.; GS 2/16.10.2017; TLMF • 2 ♀♀; same collection data; 1 Oct. 1994; Burmann et Erlebach leg.; GS 3/16.10.2017, 4/16.10.2017; TLMF • 1 ♂; prov. Verona, Albisano; alt. 450 m; 27 May–3 Jun. 1962; K. Burmann leg.; TLMF • 1 ♂; prov. Trentino, Pietramurata; alt. 250 m; mid Jun. 1978; F. Zürnbauer leg.; TLMF • 1 ♀; Verona, Mte. Maderno; alt. 250 m; mid Sep. 1963; K. Burmann leg.; TLMF • 1 ♀; Südtirol, Auer; 21 Jun. 1957; Hernegger leg.; TLMF • 3 ♂♂, 1 ♀; Südtirol, Eing. Schnalstal; alt. 700 m; mid Jun.1968; K. Burmann leg.; TLMF • 2 ♀♀; Südtirol, S Kalterer See; alt. 230 m; 26 Jun. 1990; P. Huemer leg.; TLMF • 1 ♂; same collection data; 14 Jun. 1991; TLMF • 2 ♂♂; Südtirol, Umg. Bozen, Kampenn; 26 Jun. 1991; P. Huemer leg.; TLMF • 1 ♂; Carrara; alt. 200 m; early Jun. 1979; F. Zürnbauer leg.; TLMF • 1 ♂, 1 ♀; Elba, Porto Ferroio; alt. 40 m; late May 1967; F. Zürnbauer leg.; TLMF • 1 ♂; Sardinia, Umg. Tempio, Mte. Limbara; alt. 1200 m; 27 May1973; Laubmeier, Sommerer & Witt leg.; TLMF • 1 ♂; Sardinia, Umg. Aritzo, Gennargentu; alt. 750 m; 4–5 Jun. 1973; Laubmeier, Sommerer & Witt leg.; TLMF • POLAND • 1 ♂; Poznań, Ogrody; 13 Jun. 2012; W. Kubasik leg; GS 1/15.3.2019; • CWK 1♂; Poznań, Ogrody; 19 Jun. 2012; W. Kubasik leg.; GS 2/15.3.2019; CWK • 1 ♀; Komorniki; 19 Jun. 2002; GS 1/15.3.2002; W. Kubasuk leg.; CWK • AUSTRIA • 2 ♂♂; Wien, Döbling, Cobenzl; alt. 385 m; 15 Jun. 2012; F. Lichtenberger leg.; TLMF • 3 ♂♂; Niederösterreich, 10 km SE Schwechat, Seiherwiese; 1 Jul. 1998; F. Lichtenberger leg.; TLMF • 3 ♂♂; Niederösterreich, Ebreichsdorf, Welscherhalten; 2 Jul. 1999, F. Lichtenberger leg.; TLMF • 1 ♂; Niederösterreich, Rubring; 20 Jun. 1998; F. Hofmann leg.; TLMF • 1 ♂; Niederösterreich, Amstetten, Sonnleiten; 16 Jun. 1997; H. Brandstetter leg.; TLMF • 1 ♂; Niederösterreich, Purgstall/Erlauf; 7 Jul. 1995; F. Ressl leg.; TLMF • 1 ♂; Oberösterreich, Linz, Biologiezentrum; 11 Jun. 2002; J. Wimmer leg.; GS 3771; TLMF • 2 ♂♂, 2 ♀♀; Vorarlberg, Hard, Fußach, NSG Rheindelta-Rohrspitz; alt. 397 m; 29 Jun. 1992; P. Huemer leg.; TLMF • FRANCE • 1 ♂; Alpes Maritimes, Nice, Bd. Tzarewitsch; 26 May 1971; F. Dujardin leg.; TLMF • 1 ♂; Alpes Martitimes, mt. Alban; alt. 200 m; 4 Jun. 1962; F. Dujardin leg.; TLMF • 1 ♂; Bouches du Rhone, La Ciotat; 4 May 1994; J. Nel leg.; GS 9763 TLMF • 1 ♂; Var, Massif du Pradet; J. Nel leg.; TLMF • 1 ♂; Vaucluse, Mormoiron; alt. 20 m; 6 May 2000; J. Nel leg.; TLMF • 2 ♂♂; Corse, Pinarello; alt. 10 m; early Jun. 1972; F. Zürnbauer leg.; TLMF • CROATIA • 2 ♂♂, 1 ♀; Rovinij; alt. 50 m; 10 Sep. 2002; H. Deutsch leg.; TLMF • 1 ♂; Cres isl., Stivian; 14–17 May 1996; F. Lichtenberger leg.; TLMF • GREECE • 1 ♂; Crete, Heraklion prefecture, Fodele; alt. 100 m; 25 May 2000; W. Ruckdeschel leg.; GS 5/16.10.2017; TLMF • BULGARIA • 1 ♂; Petrich district, Rupite area; 41.4620°N, 23.2583°E; alt. 200 m; 15 May 2007; B. Zlatkov leg.; GS 6/16.10.2017; NMNHS • ALBANIA • 1 ♂; Korça Region, Boboshtica Village; 40.5405°N, 20.7918°E; alt. 1220 m; 4 Jun. 2018; S. Beshkov leg.; GS 1/17.10.2018; NMNHS • MACEDONIA (Republic of Northern) • 1 ♂; Vardar River Valley, Demir Kapiya; 41.3826°N, 22.1958°E; alt. 240 m; 10 Jun. 2018; S. Beshkov & A. Nahirnic leg.; GS 2/17.10.2018; NMNHS • SPAIN • 4 ♂♂, 3 ♀♀; Catalonia, Vidreras; 6–15 Jun. 1993; J. Wimmer leg.; TLMF.

###### Diagnosis.

The wing pattern of males resembles that of *C. neglectana* and *C. eatoniana*. The male genitalia are most similar to *C. eatoniana* but the phallic process is smaller, the phallus lacks a keel, the cornuti are smaller, the valvae are more slender, and the labis is different. The females of *C. consimilana* and *C. eatoniana* externally are very similar but the genitalia (colliculum) show considerable difference, it is much larger in *C. eatoniana*, with a long process at the left side.

###### Description.

**Figure 11.**
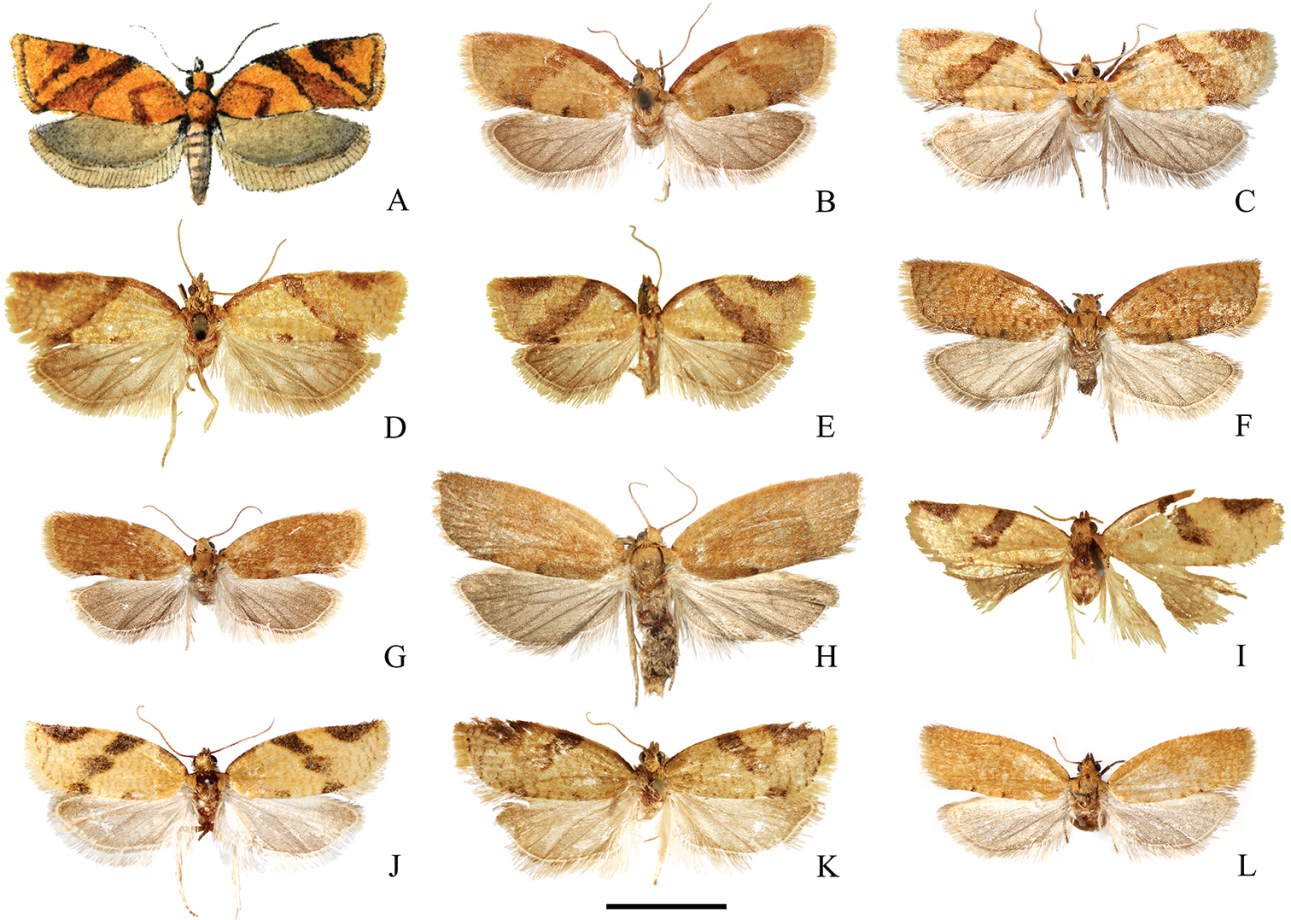
Adults of *Clepsis consimilana* species group **A–H***C. consimilana*: **A** illustration from the original description of *Tortrix consimilana***B** neotype of *T. consimilana***C** male, Bulgaria **D** lectotype of *T. unifasciana***E** Holotype of *Siclobola placida***F** male, Poland **G** female, Italy **H** female, Poland **I–L***C. eatoniana*: **I** Lectotype of *T. eatoniana***J** male, Spain **K** Lectotype of *Tortrix xylotoma* (courtesy by D. Lees and Trustees of the Natural History Museum, London) **L** female, Spain. Scale bar: 5 mm, all to scale.

**Figure 12.**
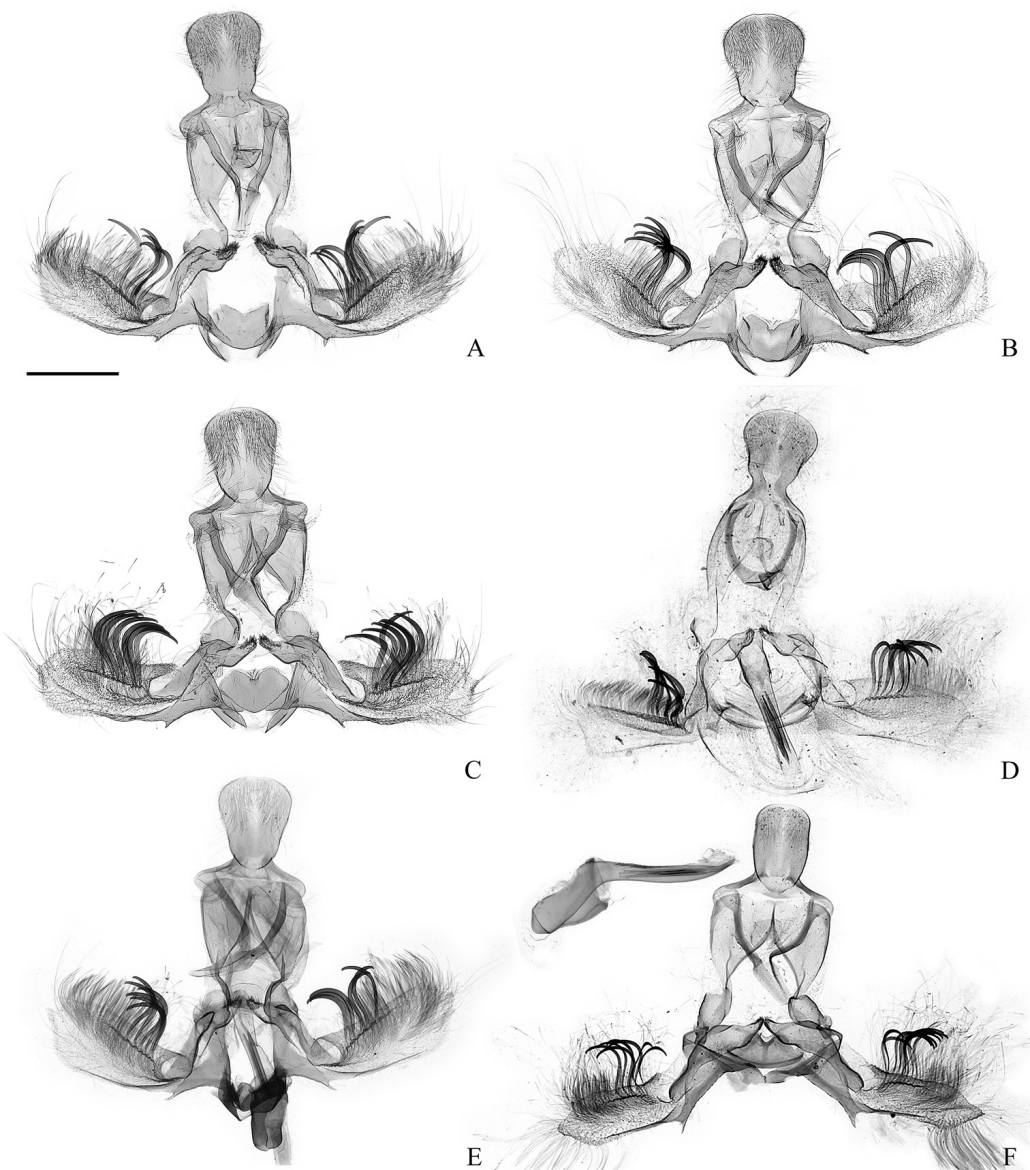
Male genitalia of the *Clepsis consimilana* species group (some without phallus) **A–E***C. consimilana*: **A***Tortrix consimilana* neotype **B***C. consimilana*, Italy **C***C. consimilana*, Albania **D***Siclobola placida* holotype **E***T. unifasciana* lectotype **F***T. eatoniana* lectotype. Scale bar: 500 μm, all to scale.

**Figure 13.**
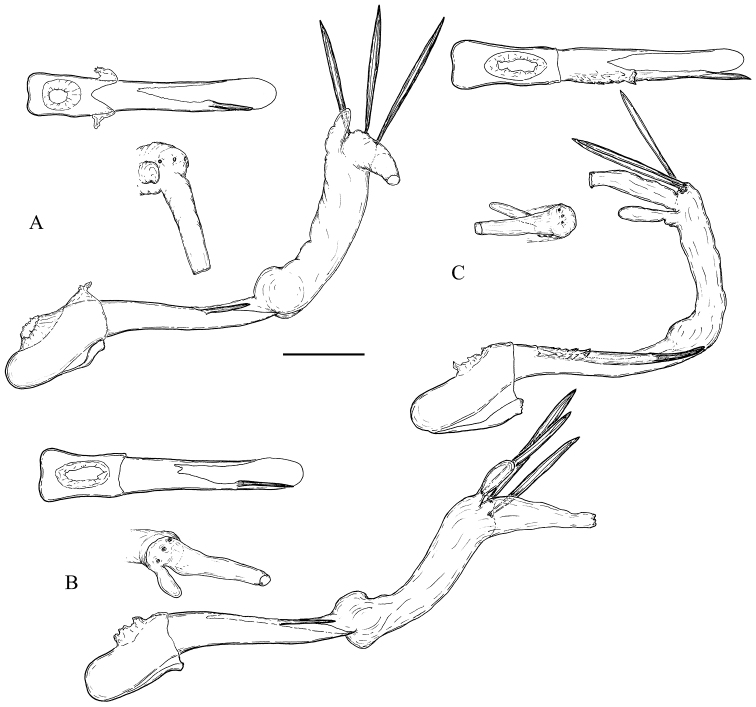
Phalli with vesicae everted of the *Clepsis consimilana* species group **A–B***C. consimilana*: **A** neotype of *Tortrix consimilana***B** specimen from Italy **C***C. eatoniana*, Spain. The phallus of each species is shown in three aspects from top to bottom: sclerotised phallic tube (without vesica) in dorsal view, tip of vesica in dorsal view and whole phallus in left view. Scale bar: 250 μm.

**Figure 14.**
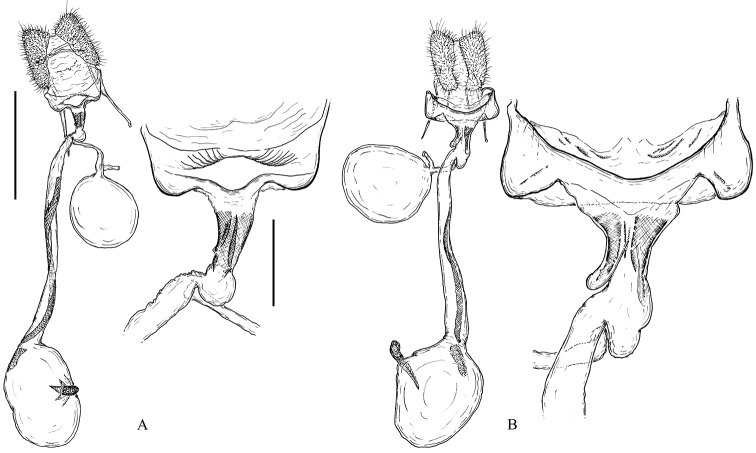
Female genitalia of the *Clepsis consimilana* species group **A***C. consimilana*, Italy **B***C. eatoniana*, Spain. The sterigma and colliculum are shown enlarged to the right of the whole genitalia. Scale bars: 1 mm (whole genitalia), 250 μm (sterigma and colliculum).

Adult. Sexual dimorphism prominent. Male (Fig. 11A–F). Head. Vertex, frons and labial palps fulvous. Antennae with scapus and pedicellus ochreous, flagellum with fulvous brown-tipped scales and numerous sensilla trichodea as long as width of flagellomeres. Thorax. Dorsally fulvous, ventrally creamy, legs brownish. Tegula fulvous. Forewing relatively wide, with length 6.3–7.8 mm (mean 7.0, *N* = 13). Costa basally convex, apically straight, costal fold extending from base to ca. 0.4–0.6 of costa (Fig. 3E). Upperside background dark yellow with rusty reticulate pattern. Markings fulvous to brown: basal blotch atrophied, more prominent at dorsum forming dark dot; median fascia often with darker borders; subapical blotch triangular, often ill-defined. Cilia concolourous with background. Underside pale brown with creamy longitudinal blotch in the distal half of costal area. Hindwings upperside monochrome grey with paler cilia, underside with creamy costal half pale grey with scattered pale brown scales and monochrome pale grey dorsal half. Forewings with variable colouration, darker or paler, sometimes with reduced markings similarly to female (Fig. 11F). Abdomen. Grey. Male genitalia (Fig. 12A–E). Uncus with variable shape and median incision depending on preparation, more or less ovoid, widened distally, rounded. Gnathos plough-shaped. Socius small, membranous. Valvae pointed dorsolaterally when mounted on slide. Costal sclerite protruded medially into large triangular labis with elongated tip (Fig. 5F, G). Apical part of sacculus ca. 1.6× longer than basal part, both forming angle of 160–165°, saccular process small, curved, thorn-shaped. Distal part of valva membranous, widened apically and protruding into small brachiola, costal edge slightly convex, with longitudinal fold on the median surface bearing row of 5–8 large modified setae. Phallus smooth, coecum medially concave, basal part curved ventrad at ca. 140°, distal part smoothly curved dorsad, with small sharp tipped lateral process as long as 0.26× distance between anterior opening and tip of phallus, slightly curved laterally and not overpassing tip of phallus. Caulis small, adjoining coecum. Vesica cylindrical, curved at angle of 100–120° to phallus, with expansion basally and finger-like sinistrodorsal diverticulum (Fig. 13A, B). Three to four deciduous aciculate, robust, weakly sinuate, slightly flattened cornuti attached ventroapically (Fig. 7D). Gonopore located at left to cornuti sockets and diverticulum. Female darker than male, with uniform forewings (Fig. 11G, H). Head. Frons, vertex and labial palps fulvous to ochreous, antennae with fulvous brown-tipped scales and sparse sensilla trichodea shorter than width of flagellomeres. Thorax dorsally ochreous, ventrally creamy, legs brownish. Forewing length 6.3–7.6 mm (mean 6.8, *N* = 3). Upperside ground colour ochreous to rusty with darker reticulated pattern and more or less atrophied ill-defined markings. Basal blotch reduced to dark spot at dorsum, median fascia more or less prominent in costal and dorsal area or completely reduced. Cilia paler than background. Underside pale brown, costal and terminal areas paler or creamy with reticulate pattern. Hindwing upperside pale grey, underside with whitish or pale grey reticulate patterned costal half and monochrome pale grey dorsal half. Abdomen. Grey. Female genitalia with papillae anales not modified (Fig. 14A). Apophyses anteriores 1.5× longer than apophyses posteriores. Sterigma widened caudad, with lateral sclerotised pockets cephalad, large excavation on the dorsal wall and wide v-shaped lamella antevaginalis. Colliculum large, with length 0.17× length of ductus bursae, slightly bent to left, funnel-shaped, with plicate longitudinal sclerotisation forming small process at left, and spherical protrusion at cranial end consisting of colourless thick cuticle. Ductus bursae long and narrow, emerging at left side of cuticular protrusion, with cestum extending along cranial 0.8× of its length. Ductus seminalis inserted dorsally at caudal end of ductus bursae. Corpus bursae ovoid, with large falcate signum with capitulum and flat signum located near end of cestum consisting of sclerotised papillae (Fig. 9D, F).

###### Preimaginal stages.

Detailed descriptions of the ovum, larvae of all instars, and pupa are provided by Sheldon (1920) and Bradley et al. (1973). The chaetotaxy of the larva is described by Swatschek (1958).

###### Molecular data

(Fig. 16). BIN: BOLD:AAC4212. The intraspecific average of the barcode region is 0.34%, the maximum distance 1.08% (p-dist) (*N* = 29). The minimum distance to the nearest neighbour, *Clepsis eatoniana*, is 2.25%.

###### Distribution

(Fig. 17). Europe, Asia Minor, Syria, European Russia, W Africa to Lebanon, Madagascar (introduced; the data come from the type specimen only), North America (introduced) (Bradley et al. 1973, Razowski 2002).

###### Ecology.

Moths are on the wing from May to October in shrubby habitats. The larvae feed on dead or withered leaves of *Crataegus*, *Malus*, *Carpinus*, *Polygonum*, *Hedera*, *Lonicera*, *Ligustrum*, *Syringa*, and overwinter in the third instar (Bradley et al. 1973).

###### Remarks.

The collection of Jakob Hübner was acquired by Vincenz Abbate Edler von Mazzola in the early part of the 19^th^ Century, and the European material was deposited in the “Hof-Naturalien-Kabinett” in 1823 where most of the material was destroyed by fire during the Vienna Rebellion of 1848 (Calhoun 2003, Gilligan and Wright 2013). Despite a personal search by PH we found no potential type material and conclude that the syntypes are lost or destroyed. As the boundaries of this taxon are revised, designation of a neotype is necessary to preserve the stability of nomenclature. The type locality is presumably Europe, as indicated by the title of the original description (Hübner 1817) and material may have originated from Germany as many other species described by Hübner. Here we designate as neotype a male specimen with an everted vesica and preserved cornuti, and wing pattern resembling the illustration in the work by Hübner (1817: pl. 38, fig. 239). Though not very detailed, this painting demonstrates some important differences with *C. consimilana* sensu auctt., namely the shape of basal fascia and costa, and questions the interpretation of the taxon. The painting either refers to another representative of the tribe Archipini or may be a result of an artistic decision. Without studying the type material any interpretation of the figure may be incorrect, therefore we prefer to preserve the present-day concept for *C. consimilana*. Obraztsov (1955) considered *consimilana* as a probable synonym of *unifasciana*. Bradley and Martin (1956) established the name *consimilana* as having priority over *unifasciana*, though they did not state explicitly this nomenclatorial act. We found support for this synonymy as the lectotype of *Tortrix unifasciana* appears conspecific with the neotype of *C. consimilana*. Razowski (1979) synonymised *Tortrix eatoniana* with *C. consimilana* (see below); this is not justified in our opinion. Some taxa from the synonymic list were not examined by us but, minding the minimal genetic diversity throughout Europe, we consider all of them correctly synonymised with *C. consimilana*. Cacoecia unifasciana var. semiana was erroneously regarded as a synonym of *Clepsis consimilana* (see above).

##### Clepsis eatoniana

Taxon classificationAnimaliaLepidopteraTortricidae

(Ragonot, 1881) stat. rev.

B9BD8C9E-92C1-59D8-BC8D-950C3C830553

Tortrix eatoniana Ragonot, 1881: 231 (Portugal).
Tortrix xylotoma Meyrick, 1891: 13 (Algeria) syn. nov.
Clepsis razowskii Gastón, Vives & Revilla, 2017: 691 (Spain) syn. nov.
non Clepsis consimilana (Hübner, 1817)

###### Material examined.

Lectotype ♂ by designation of Razowski (1979), pinned, with 7 labels: “Eatoniana / Rag. n. sp. / Portugal” [handwritten; the same label on backside:] “port Olivaes / Port. [ugal] / 24.4.80” “*Tortrix / eatoniana* Rag. / Ent. month. Mag., / 1881, 17, p. 231” [handwritten] “Mus. Paris / coll. Ragonot / 15997” [printed and handwritten] “Type” [red printed] “communiqué / á M. J. Kennel” [handwritten] “Genitalia ♂ / P. Viette / prép. No. 3699” [printed] “1901 / coll. E. L. Ragonot / Muséum Paris” [printed]; male genitalia on a slide with two labels: “♂ / *Tortrix* / *eatoniana* / Rag. / Type” “Prép. P. Viette / # 3699 / Muséum / Paris / Type” [both handwritten, with red border].

PORTUGAL • 1 ♂; Lisbon, Olivais; 24 Apr. 1880; Ragonot leg.; GS 3699; MNHN.

Lectotype ♂ of *Tortrix xylotoma* by designation of Razowski (1979), pinned, with 7 labels: “Bougie / Algeria / 25/4/90” [handwritten] “Tortrix / xylotoma / Meyr[ick] / Type ♂” [handwritten and printed] “Lecto- / type” [circular with violet border] “Type” [circular with red border] “♂ genitalia on / slide 6.IV.1949 / J.F.G.C. 9364” [printed and handwritten] “Meyrick coll. / B. M. 1938–290” “NHMUK010219594 [QR code]”.

ALGERIA • 1 ♂; Béjaïa; 25 Apr. 1890; Meyrick leg.; BMNH NHMUK010219594.

Other material: SPAIN • 8 ♂♂, 1 ♀; Valencia, El Saler, Albufera ; 39.3278°N, -0.3078°W; alt. 5 m; 18 May 2004; P. Huemer leg.; GS 1/18.10.2017, 2/18.10.2017, 3/18.10.2017; TLMF • 5 ♂♂, same collection data; 8 Sep. 2005; P. Huemer leg.; TLMF • 1 ♂, 1 ♀; Valencia, 5 km NE Albufera, Sierra de Crevillente; alt. 450 m; 26 May 2004; P. Huemer leg.; TLMF • 1 ♀; Valencia, Santa Pola, Playa del Pinet; 38.1585°N, -0.6256°W; alt. 5 m; 5Sep. 2005; P. Huemer leg.; GS 4/18.10.2017; TLMF.

###### Diagnosis.

The wing pattern in males resembles those of *C. trivia*, *C. acclivana*, and *C. consimilana*. *Clepsis eatoniana* differs from *C. consimilana*, by the more yellowish instead of fulvous forewing ground colour. The most characteristic feature in the external morphology of *C. eatoniana* is the absence of a forewing costal fold, in contrast to *C. consimilana*. The male genitalia are similar to those in *C. consimilana* but the valva is more slender, elongate distally, and the modified large setae are more numerous and more slender. The phallus is adorned with spines, with the keel and terminal process overpassing its tip; in *C. consimilana* the phallus lack lateral spines and a keel, and the phallic process is shorter. The caulis in *C. eatoniana* is larger, the vesica is bent at nearly a right angle to the phallus, its diverticulum has different location, and the cornuti are more slender and smoother. Females of *C. eatoniana* are not distinguishable externally from the females of *C. consimilana* but differ from *C. striolana* by the presence of two brown dots on the forewing. The female genitalia of *C. eatoniana* are similar to those in both the *C. neglectana* and *C. consimilana* species groups, but the protrusion on the right side of the colliculum is much larger and elongated, and the sterigma has larger lateral pockets.

###### Description.

**Figure 15.**
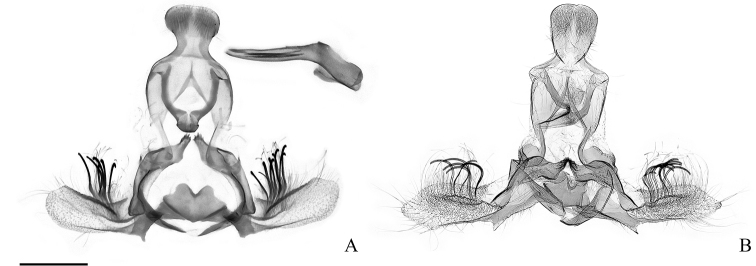
Male genitalia of the *Clepsis consimilana* species group: *C. eatoniana***A***Tortrix xylotoma* lectotype (courtesy by D. Lees and Trustees of the Natural History Museum, London) **B***C. eatoniana*, Spain. Scale bar: 500 μm, all to scale.

Adult. Sexual dimorphism prominent. Male (Fig. 11I–K). Head. Vertex fulvous, frons and labial palps brown. Antennae with brown scales on scapus and fulvous scales on pedicellus and flagellum, and with numerous sensilla trichodea as long as width of flagellomeres. Thorax. Fulvous dorsally and creamy ventrally, legs brownish. Tegula fulvous with brown costal margin. Forewing with length 6.8–7.4 mm (mean 7.1, *N* = 3), elongated, costa convex at basal half, slightly sinuate apically. Costal fold lacking (Fig. 3F). Upperside background dark yellow with fulvous reticulate pattern more prominent in the paler subterminal area, cilia concolourous or paler. Basal blotch atrophied, with remnants of darker scales at costa and dorsum. Median fascia brown with lead refractive tint, almost interrupted at middle of median cell by yellowish scales, often ceasing at vein CuA. Subapical blotch brown, triangular, more or less well defined. Underside pale grey-brown with creamy longitudinal blotch in distal half of costal area. Specimens with paler and darker forewings and incomplete median fascia observed. Hindwing upperside monochrome pale grey with paler cilia, underside whitish with scattered pale grey-brown scales. Abdomen. Grey. Male genitalia (Fig. 12F, 15). Uncus variably shaped, more or less trapezoidal, slightly widened distally, rounded, with slightly convex distal edge which may look incised if excessive pressure is applied on coverslip. Gnathos plough-shaped. Socius small, membranous. Valvae pointed dorsolaterally when mounted on slide. Costal sclerite wide, protruded medially into large triangular labis with elongated spinulate tip (Fig. 5H). Basal and apical part of sacculus of nearly equal length, both forming angle of 150–160°, saccular process large, flat, triangular. Distal part of valva membranous, comparatively small, narrow, with parallel costal and saccular margins, apically triangular, without distinct brachiola, with longitudinal fold on the median surface bearing row of 8–12 large modified setae. Apical part may look as brachiola due to deformation during preparation. Phallus robust, coecum medially concave, basal part curved ventrad at ca. 140°, distal part smoothly curved dorsad, with wide keel on left side grading into large sharp tipped lateral process as long as 0.23× distance between anterior opening and tip of phallus, slightly curved laterally and dorsally, overpassing phallic tip. Several more or less prominent spines pointed caudad located at basal part of keel. Caulis large, widely separated from coecum. Vesica cylindrical, curved at 80–90° to phallus, with small expansion basally and fingerlike dorsal diverticulum (Fig. 13C). Three deciduous, slender, straight cornuti attached ventroapically (Fig. 7E). Gonopore located in straight line with cornuti sockets and diverticulum. Female darker than male (Fig. 11L). Head. Vertex rusty, frons and labial palps brown. Whole antennae with brown scales, sensilla trichodea sparse, shorter than width of flagellomeres. Thorax. Rusty dorsally and creamy ventrally, legs brownish. Tegula rusty with brown costal margin. Forewing length 6.7 mm (n = 1), with shape as in males, upperside uniformly rusty with ill-defined reticulate pattern more distinct in distal half and two brown dots at dorsum, underside mainly creamy with scattered pale brown scales, denser in middle of wing. Hindwing upperside grey brown, underside creamy with darker dorsal part. Abdomen grey.

Female genitalia (Fig. 14B). Papillae anales not modified. Apophyses anteriores 1.1× longer than apophyses posteriores. Sterigma relatively wide, widened cephalad, with large lateral sclerotised pockets, large excavation on dorsal wall and wide v-shaped median part of lamella antevaginalis. Colliculum large, with length 0.25× length of ductus bursae, asymmetrical, funnel-shaped, with well-developed sclerotisation and two evaginations: very large one at right and small one at left, both consisting of colourless thick cuticle. Ductus bursae long and narrow, emerging at left between out-pocketings, with cestum extending along cranial 0.7× of its length. Ductus seminalis inserted dorsally at caudal end of ductus bursae. Corpus bursae ovoid, with large falcate signum with capitulum and flat signum consisting of sclerotised papillae located near end of cestum (Fig. 9E).

Preimaginal stages unknown.

###### Molecular data

**Figure 16.**
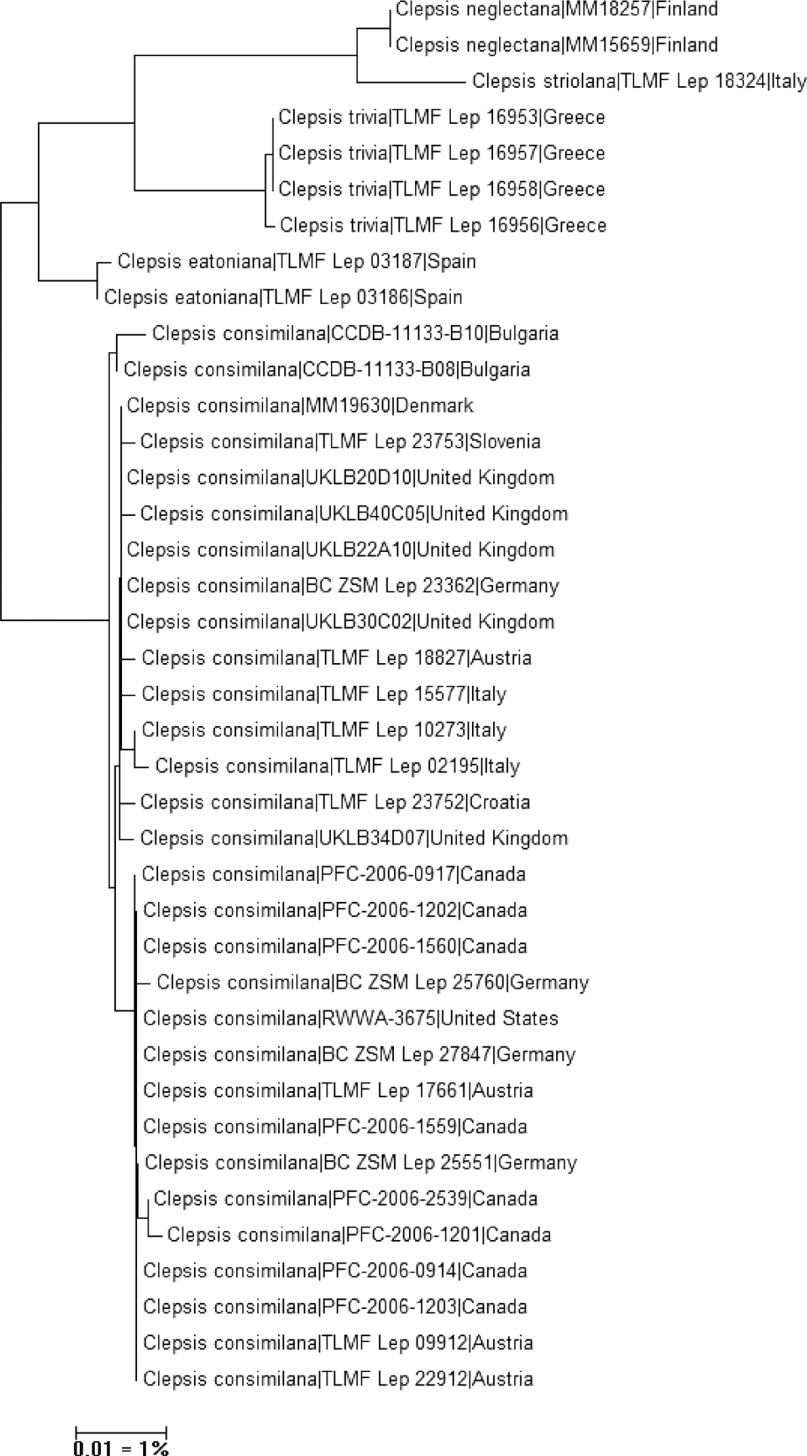
Neighbour-joining tree of *Clepsis neglectana* and *C. consimilana* species groups (Kimura 2 parameter, built with MEGA6; cf. Tamura et al. 2013). Note: the scale bar only applies to internal branches between species. The width of the triangles represents the sample size, and the depth the relative genetic variation within the cluster (2× scale bar). Source: DNA Barcode data from BOLD (Barcode of Life Database, cf. Ratnasingham and Hebert 2007).

(Fig. 16). BIN: BOLD:AAJ1025. The intraspecific average of the barcode region is 0.09%, the maximum distance 0.15% (p-dist) (*N* = 2). The minimum distance to the nearest neighbour *Clepsis consimilana* is 2.25%.

###### Distribution

**Figure 17.**
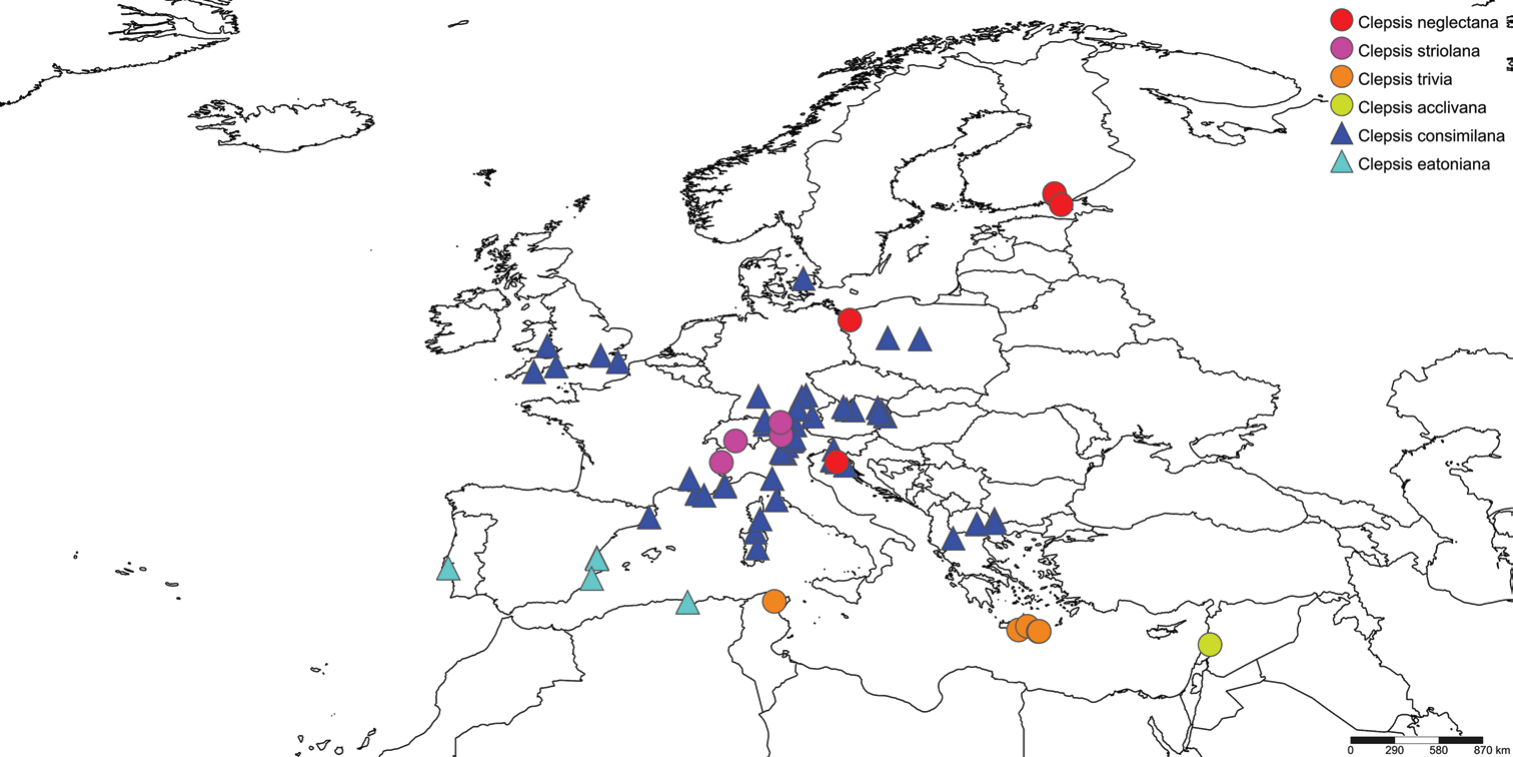
Distribution map of examined material of *Clepsis neglectana* and *C. consimilana* species groups. Map created with SimpleMappr (http://www.simplemappr.net).

(Fig. 17). Europe. Portugal: Lisbon, Olivais; Ponte de Mucela (Ponte de Morcellos) (Ragonot 1881); Lusitania; Spain: Cadiz, Granada, Malaga, Sevilla, Zaragoza; possibly France (Gastón et al. 2017), Valencia. Africa: Algeria.

###### Ecology.

The moths were collected in macchie habitat (Gastón et al. 2017) from April to the first half of September. The larval host plant is unknown.

###### Remarks.

The species was described by Ragonot (1881) after two males from Portugal. He emphasised its resemblance with *C. consimilana*. Razowski (1979) considered it conspecific with *C. consimilana*, probably relying on the superficial similarity of the modified setae of the valvae of these two taxa. The female remained unstudied until Gastón et al. (2017) described it again under the name *C. razowskii*. *Tortrix xylotoma* was sunk into *C. neglectana* by Razowski (1979). This synonym is not justified as the genitalia of the male lectotype (illustrated also by Clarke 1958: pl. 109) rather resemble *C. eatoniana* regarding the shape of labis, presence of modified setae on the valva, and morphology of the phallus. The preparation of the genitalia is poor, therefore the shape of uncus and valvae looks unusual. The wing pattern fits with *C. eatoniana*. After comparison of material from Spain, the lectotype of *T. eatoniana*, the lectotype of *T. xylotoma*, the original description of *C. razowskii*, and numerous photographs of specimens identified as *C. razowskii* by the authors of the latter taxon, we concluded that all these specimens are conspecific and the present valid name of the species should be *C. eatoniana*.

## Discussion

The taxa treated in the present paper are not easily distinguished from each other because of prominent sexual dimorphism in some of them, variability in wing pattern and considerable similarity in the genitalia. The subtle differences in both male and female (when available) genitalia, secondary sex characters (male forewing costal fold) and barcode gap between the populations support presence of more than the two taxa currently recognised. The most defining characters are found in the genitalia. Two groups of taxa can be recognised: those with large modified setae on the median surface of the valvae in males, and those without such setae. Apparently, this was the main criterion for prior synonymisation resulting in only two species: *C. consimilana* (with setae) and *C. neglectana* (without setae). Further scrutinising of the genital characters revealed existence of several taxa, which can be treated as species. Here we propose two species groups named after the oldest described taxon in each of them. They should not be confused with the groups proposed by Razowski (1979) for subgeneric divisions of *Clepsis*, which appear hierarchically superior to our groups.

Since recently collected material for mtDNA sequencing was available for European populations only, the status of some taxa of the *C. neglectana* species group remains unresolved. *Clepsis severana* and *C. semiana* appear to be closely related considering the genital morphology, and their synonymy with some of the remaining species of the group cannot be excluded. They are known only from their holotypes (by monotypy), and moreover, the morphology of the vesica is inaccessible because of the prolonged embedding in microscope slide media (*C. semiana*) or the genitalia slide is missing (*C. severana*). On the other hand, each of them demonstrates some differences with all remaining members of the group, which seemingly are not lesser than those between well separated taxa as *C. neglectana* and *C. trivia*. *Tortrix xylotoma* is certainly a synonym of a member of the *C. consimilana* species group, namely *C. eatoniana*, but not of *C. neglectana* as it was treated up to now. The status of *C. acclivana* is more or less controversial because of lacking DNA barcode sequences, but the two male specimens with practically identical morphology, different to all the other taxa convinced us that they represent a distinct species.

The Cretan population of *C. trivia* is well separated genetically and morphologically from the other European species (*C. neglectana* and *C. striolana*), but demonstrates certain morphological similarity with the other Mediterranean taxon, *C. acclivana*, and is probably related to it. It should be emphasised that the Cretan specimens were assigned to *C. trivia* solely on male genitalia morphology without studying the vesica of the holotype and its DNA barcode and it may still represent a separate taxon.

The barcode distance between *C. neglectana* and *C. striolana* is comparatively small. The genital morphology of these two taxa also demonstrates considerable similarity, but they are well distinguished by wing pattern and costal fold. Apparently, *C. striolana* has a range limited in the region of the Alps. It can be assumed that these species had been isolated recently, maybe only after the last glacial period, and even continuing gene flow cannot be dismissed. Some taxa still in synonymy with *C. neglectana* may turn out to be *species bona* as well, e.g., *Tortrix dorana* from Eastern Kazakhstan.

The *C. consimilana* species group encompasses only two species and is separated from the *C. neglectana* group by considerable morphological and genetic gaps. The species have strikingly disproportional distribution ranges: *C. eatoniana* appears to be a Western-Mediterranean species distributed in the Iberian Peninsula and Algeria, and possibly part of France, but *C. consimilana* is distributed throughout the rest of Europe, part of Asia and has been introduced to the tropics (Madagascar) and North America. Some morphological variability in *C. consimilana* (the position of cornuti and other minor male genitalia characters) somewhat contradicts the genetic invariance within this species in Europe.

The overreliance or underestimation of certain genital characters can cause incorrect taxonomic interpretations, as it is demonstrated by the above mentioned taxonomic problems. Traditionally, the shape of uncus, gnathos and valvae is widely used in taxonomy of Lepidoptera, but our experience with other groups as well (Zlatkov and Huemer 2017) proved that these characters must be treated cautiously. Being complex three-dimensional structures, their visible shape depends on several factors: fixation before embedding in a permanent medium, thickness of the medium layer and pressure applied on the coverslip. The shape of uncus and distal part of valva are especially prone to deformation in the species treated here. Some relatively flat sclerites were found to be less affected and hence more useful for taxonomical purposes, e.g., the sacculus and in lesser extent the costal sclerite, including the labis, of the valva. The morphology of the vesica and phallus is believed to be very informative but must be studied before mounting on a slide. Moreover, these structures are considerably simplified in the taxa considered here and provide a relatively small number of characters. The female genitalia can also provide many characters but only in a comparatively limited region: the colliculum and part of the sterigma. In fact, these are the structures which interact directly with the male genitalia. It can be assumed that the distal part of the phallus is inserted into the colliculum and its lateral process is accommodated into the transparent cuticular extension at the right cephalic end, therefore they should be studied in comparative aspect to obtain additional taxonomical information. Some taxonomic problems in the *C. neglectana* and *C. consimilana* species groups had apparently ensued from ignoring female genitalia morphology or simply the lack of female specimens.

## Supplementary Material

XML Treatment for Clepsis neglectana

XML Treatment for Clepsis striolana

XML Treatment for Clepsis trivia

XML Treatment for Clepsis acclivana

XML Treatment for Clepsis severana

XML Treatment for Clepsis semiana

XML Treatment for Clepsis consimilana

XML Treatment for Clepsis eatoniana
